# Mifepristone alone and in combination with scAAV9-*SMN1* gene therapy improves disease phenotypes in *Smn*^*2B/-*^ spinal muscular atrophy mice

**DOI:** 10.1038/s41598-025-24050-3

**Published:** 2025-11-17

**Authors:** Emma R. Sutton, Eve McCallion, Joseph M. Hoolachan, Özge Çetin, Paloma Pacheco-Torres, Saman Rashid, Sihame Bouhmidi, Katie Haynes, Lauren Churchill, Taylor Scaife, Helena Chaytow, Yu-Ting Huang, Stephanie Duguez, Bernard L. Schneider, Thomas H. Gillingwater, Maria Dimitriadi, Melissa Bowerman

**Affiliations:** 1 School of Medicine, Keele University, Staffordshire, UK; 2 School of Health, Medicine and Life Sciences, University of Hertfordshire, Hertfordshire, UK; 3 School of Life Sciences, Keele University, Staffordshire, UK; 4 Centre for Discovery Brain Sciences, Edinburgh Medical School: Biomedical Sciences, University of Edinburgh, Edinburgh, UK; 5 Euan MacDonald Centre for Motor Neuron Disease Research, University of Edinburgh, Edinburgh, UK; 6 Personalised Medicine Centre, School of Medicine, Ulster University, Derry, UK; 7 Bertarelli Platform for Gene Therapy, Swiss Federal Institute of Technology (EPFL), Lausanne, Switzerland; 8 Wolfson Centre for Inherited Neuromuscular Disease, RJAH Orthopaedic Hospital, Oswestry, UK

**Keywords:** Metabolism, *Klf15*, Skeletal muscle, Spinal muscular atrophy, Mifepristone, Combinatorial therapy, Molecular biology, Diseases, Drug discovery

## Abstract

Spinal muscular atrophy (SMA) is a neuromuscular disease caused by deletions or mutations in the *survival motor neuron 1* (*SMN1*) gene. SMA is characterised by alpha motor neuron loss in the spinal cord and subsequent muscle atrophy. There are currently three approved SMN-directed therapies for SMA patients. While these therapies have transformed what was once a life-limiting condition into one that can be managed and even improved, they are unfortunately not cures, highlighting the need for additional supporting second-generation therapies. These should not only target the neuromuscular system but also peripheral and metabolic perturbations that are present in both SMA models and patients. Krüppel-like factor 15 (Klf15) is a transcription factor that maintains metabolic homeostasis, is involved in the glucocorticoid-glucocorticoid receptor (GR) signalling pathway and is dysregultated in several peripheral and metabolic tissues in SMA mice. Here, we used murine and human cellular models as well as SMA mice and *Caenorhabditis Elegans (C. elegans)* to assess the therapeutic potential of reducing *Klf15* activity with mifepristone, a glucocorticoid antagonist, combined with a SMN-targeted gene therapy. We report that mifepristone reduces *Klf15* expression across several in vitro models, ameliorates neuromuscular pathology in SMA *smn-1(ok355) C. elegans* and improves survival of SMA *Smn*^*2B/-*^ mice. Furthermore, we show that combining mifepristone with an approved SMN-directed gene therapy (scAAV9-*SMN1*) results in improved tissue- and sex-specific responses to treatment. Our study demonstrates that a multi-tissue targeting SMN-independent drug, alone and in combination with an approved SMN-dependent therapy, has the potential to improve SMA disease pathology.

**Supplementary Information:**

The online version contains supplementary material available at 10.1038/s41598-025-24050-3.

## Introduction

Spinal muscular atrophy (SMA) is a neuromuscular disorder characterised by the loss of alpha motor neurons in the anterior horn of the spinal cord and subsequent muscle atrophy^1^. In addition to defects within the neuromuscular system, emerging studies have reported peripheral pathologies and metabolic perturbations in both human patients and mouse models^2–4^. SMA is caused by a significant depletion but not complete loss of the survival motor neuron (SMN) protein^5,6^. This is due to loss-of-function deletions and/or mutations in the *survival motor neuron 1* (*SMN1*) gene that are partially compensated by the presence of a second gene, the *survival motor neuron 2* (*SMN2*) gene, that is capable of producing ~ 10% of fully functional SMN protein^1,7^.

Ground-breaking and approved *SMN1* and *SMN2*-directed therapies for SMA (Spinraza, Zolgensma, Risdiplam) provide sustained improvement in motor function and increase lifespan of many patients, but these therapeutics are currently not a cure^8^. As a result, the field is now looking beyond SMN and the neuromuscular system for additional contributors to pathology that may be targeted to provide additional therapeutic benefits^9^. Peripheral pathologies in tissues such as the liver, heart, pancreas and skeletal muscle have been repeatedly reported in SMA mouse models and patients^4,10–12^. Interestingly, these tissues play an important role in maintaining systemic energy homeostasis and their intrinsic defects in SMA could have significant consequences on whole-body metabolic homeostasis. Indeed, perturbations in fatty acid, amino acid and glucose metabolism have been observed in SMA mouse models and patients^3,10,13,14^. The fact that dietary supplementation improves lifespan of SMA mice^15–17^ further supports the hypothesis that metabolic perturbations contribute to SMA pathology. More recently, we also demonstrated that providing an amino-based formula to children with SMA that had received an *SMN2*-directed treatment, significantly reduced their persisting gastrointestinal issues^18^. Significant research also demonstrates additional peripheral comorbidities commonly reported in patients with SMA such as Metabolic Dysfunction-Associated Steatotic Liver Disease (MASLD; formally known as NAFLD) and type 1/2 diabetes^4,19^. These studies highlight the importance and relevance of tackling peripheral and metabolic phenotypes in SMA with a combination of approved SMN-directed treatments and second-generation interventions^20,21^.

An interesting and potential SMN-independent target is the transcription factor Krüppel-like factor 15 (Klf15)^3^. Transcriptional regulation is a main control mechanism of metabolic homeostasis and Klf15, a member of the zinc finger transcription factors, has an overarching influence on metabolic processes including those that are perturbed in SMA models and patients (e.g. fatty acid, amino acid and glucose metabolism)^22–25^. The rhythmic expression of *Klf15* over a 24 h period is modulated by the circadian secretion of glucocorticoids (GCs) and activity of the glucocorticoid receptor (GR)^26,27^. Klf15 modulates several metabolic pathways, including the utilization of the branched-chain amino acids (BCAAs) valine, leucine and isoleucine^24^. We have previously shown an aberrant activity of the *Klf15*-GC-GR-BCAA pathway in serum and metabolic tissues from severe *Smn*^*-/-*^*;SMN2* and intermediate *Smn*^*2B/-*^ SMA mice, whereby the levels of *Klf15* and GCs were elevated and BCAAs depleted^3^. Importantly, modulation of the pathway by daily oral administration of BCAAs to *Smn*^*-/-*^*;SMN2* mice led to significant improvements in weight and survival^3^. While a direct link between SMN and Klf15 has currently not been demonstrated, we have previously reported the age- and tissue-dependent diurnal expression of the *Smn* gene alongside core clock genes in metabolic tissues of SMA mice during disease progression^28^, suggesting a potential functional relationship between SMN, peripheral circadian rhythms and metabolic homeostasis.

The GC-GR-Klf15-BCAA pathway therefore contributes to SMA pathogenesis and could be a potential therapeutic target to alleviate peripheral and metabolic pathologies in SMA. As modulating BCAA metabolism, a downstream component of the GC-GR-Klf15 signalling cascade, provided significant improvements in SMA mice, it is possible that targeting an upstream effector may lead to a more direct and specific modulation of the GC-Klf15 pathway and thus, greater benefits. In this study, we therefore examined the therapeutic potential of mifepristone, a commercially available GR antagonist, for the treatment of SMA, alone and in combination with an SMN-dependent gene therapy. Furthermore, mifepristone appeared in a list of therapeutic candidates in our recently published study combining multi-omics and bioinformatics approaches to identify repurposed drugs for the management of muscle pathologies and SMA^29^. Interestingly, mifepristone is currently being evaluated in clinical trials for several conditions including neurodegenerative and metabolic diseases such as cancer (ClinicalTrials.gov ID NCT03225547), Cushing’s syndrome (ClincalTrials.gov ID NCT00569582), Type 2 diabetes (ClinicalTrials.gov ID NCT05772169) and Alzheimer’s disease (ClinicalTrials.gov ID NCT00105105)^30^.

In this study, we set out to determine the therapeutic potential of mifepristone in SMA. Our initial assessments found that mifepristone’s ability to reduce GC-induced *Klf15* expression was dependent upon cell type and differentiation state across representative immortalized cells lines for different metabolic tissue types (muscle, brown adipose tissue (BAT) and liver). Importantly, we found that mifepristone treatment across SMA animal models, including severe *Smn*^*-/-*^*;SMN2* SMA mice, milder *Smn*^*2B/-*^ SMA mice and a severe *C. elegans* SMA model, significantly improved disease phenotypes such as survival, muscle size and neuromuscular function. Finally, we assessed the potential synergistic activity of combining mifepristone with an *SMN1* gene-based therapy and observed tissue- and sex-specific effects. Overall, our study supports the relevance of using GC-antagonist drugs as secondary therapies alongside SMN-dependent treatments for targeting both neuromuscular and metabolic pathologies in SMA.

## Results

### Mifepristone reduces *Klf15* expression in cellular models of metabolic tissues

We initially assessed mifepristone activity in immortalised cells that reflect metabolic tissues in which we had reported increased expression of *Klf15*^3^: the C2C12 cell line for skeletal muscle, the 3T3-L1 cell line for brown adipose tissue (BAT) and the FL83B cell line for liver tissue^31–33^.

To model the increase in *Klf15* expression seen in tissues of SMA mice^3^, we used a synthetic glucocorticoid, dexamethasone, to induce *Klf15* expression. To determine the optimal dosing regimen for maximal expression of *Klf15*, we treated cells with different concentrations of dexamethasone (1, 5 and 10 μM) across different time points (4, 8 and 24 h) (Supplementary Fig. 1A–C). We therefore determined that the optimal treatment regimens were: 10 μM for 24 h for both differentiated C2C12 myotubes and FL83B hepatocytes as well as 10 μM for 4 h for 3T3-L1 brown adipocytes (Supplementary Fig. 1A–C).

For each cell line, we assessed the expression of both GR isoforms, whereby GRα is the main mediator of GCs while GRβ inhibits GRα and induces GC resistance^34,35^. We then determined mifepristone’s ability to reduce *Klf15* expression by adding it (1, 5 and 10 μM) before or after the previously determined optimal dexamethasone treatment. In addition, we evaluated the effect of mifepristone on cell death and proliferation, using the lactate dehydrogenase (LDH)-Glo™ assay and a Bromo-2-deoxyuridine (BrdU) colorimetric assay, respectively.

**Fig. 1:**
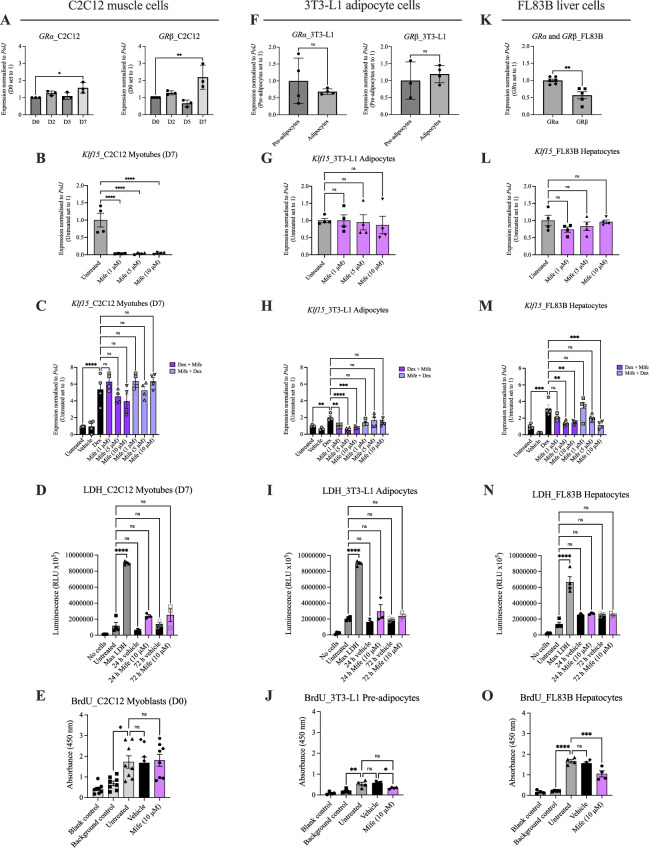
Mifepristone reduces *Klf15*-induced expression in various cellular models of metabolically active tissues. (**A**), GRα and GRβ isoform expression in C2C12 cells over a 7 day (D7) differentiation period. Data are mean ± SEM, N = 3 experimental repeats (3–4 wells/repeat), one-way ANOVA, *P < 0.05, **P < 0.01. (**B**), *Klf15* expression following mifepristone treatment (1, 5 or 10 μM) for 8 h in C2C12 myotubes (D7). Untreated cells served as control. Data are mean ± SEM, N = 4 experimental repeats (3–4 wells/repeat), one-way ANOVA, ****P < 0.0001. (**C**), *Klf15* expression following mifepristone (1, 5 or 10 μM) for 2 h followed by dexamethasone (10 μM) for 8 h or vice versa in C2C12 myotubes (D7). Untreated and vehicle-treated cells served as controls. Data are mean ± SEM, N = 4 experimental repeats (3–4 wells/repeat), one-way ANOVA, ns = not significant, ****P < 0.0001. (**D**), LDH assay (cell death assay) in C2C12 myotubes (D7) following 24 h and 72 h treatment with mifepristone (10 μM). No cells, untreated cells and max LDH served as controls. Data are mean ± SEM, N = 3–4 experimental repeats (3–4 wells/repeat), one-way ANOVA, ns = not significant, ****P < 0.0001. (**E**), BrdU assay (proliferation assay) in C2C12 myoblasts (D0) following 72 h treatment with mifepristone (10 μM). Blank, background and untreated cells served as controls. Data are mean ± SEM, N = 8 experimental repeats (3–4 wells/repeat), one-way ANOVA, ns = not significant, *P < 0.05. (**F**), GRα and GRβ isoform expression in 3T3-L1 pre-adipocytes and adipocytes. Data are mean ± SEM, N = 3–4 experimental repeats (3–4 wells/repeat), unpaired *t-test*, ns = not significant. (**G**), *Klf15* expression following mifepristone treatment (1, 5 or 10 μM) for 4 h in 3T3-L1 adipocytes. Untreated cells served as control. Data are mean ± SEM, N = 4 experimental repeats (3–4 wells/repeat), one-way ANOVA, ns = not significant. (**H**), *Klf15* expression following mifepristone (1, 5 or 10 μM) for 2 h followed by dexamethasone (10 μM) for 4 h or vice versa in 3T3-L1 adipocytes. Untreated and vehicle-treated cells served as controls. Data are mean ± SEM, N = 4 experimental repeats (3–4 wells/repeat), one-way ANOVA, ns = not significant, **P < 0.01, ***P < 0.001, ****P < 0.0001. (**I**), LDH assay (cell death assay) in 3T3-L1 adipocytes following 24 h and 72 h treatment with mifepristone (10 μM). No cells, untreated cells and max LDH served as controls. Data are mean ± SEM, N = 3–4 experimental repeats (3–4 wells/repeat), one-way ANOVA, ns = not significant, ****P < 0.0001. (**J**), BrdU assay (proliferation assay) in 3T3-L1 pre-adipocytes following 72 h treatment with mifepristone (10 μM). Blank, background and untreated cells served as controls. Data are mean ± SEM, N = 4 experimental repeats (3–4 wells/repeat), one-way ANOVA, ns = not significant, *P < 0.05, **P < 0.01. (**K**), GRα and GRβ isoform expression in FL83B cells. Data are mean ± SEM, N = 5–6 experimental repeats (3–4 wells/repeat), unpaired *t-test*, *P* = 0.0021. (**L**), *Klf15* expression following mifepristone treatment (1, 5 or 10 μM) for 24 h in FL83B cells. Untreated cells served as control. Data are mean ± SEM, N = 4 experimental repeats (3–4 wells/repeat), one-way ANOVA, ns = not significant. (**M**), *Klf15* expression following mifepristone (1, 5 or 10 μM) for 2 h followed by dexamethasone (10 μM) for 24 h or vice versa in FL83B cells. Untreated and vehicle-treated cells served as controls. Data are mean ± SEM, N = 4 experimental repeats (4 wells/repeat), one-way ANOVA, ns = not significant, **P < 0.01, ***P < 0.001. (**N**) LDH assay (cell death assay) in FL83B cells following 24 h and 72 h treatment with mifepristone (10 μM). No cells, untreated cells and max LDH served as controls. Data are mean ± SEM, N = 3–4 experimental repeats (3–4 wells/repeat), one-way ANOVA, ns = not significant, ****P < 0.0001. (**O**) BrdU assay (proliferation assay) in FL83B cells following 72 h treatment with mifepristone (10 μM). Blank, background and untreated cells served as controls. Data are mean ± SEM, N = 4 experimental repeats (3–4 wells/repeat), one-way ANOVA, ns = not significant, ***P < 0.001, ****P < 0.0001.

In differentiated C2C12 myotubes (D7), we found a significant increase in the expression of both *GRα* and *GRβ* isoforms compared to proliferating myoblasts (D0) (Fig. 1A), suggesting differential glucocorticoid sensitivities throughout muscle development. Next, we investigated the ability of mifepristone to reduce dexamethasone-induced *Klf15* expression in C2C12 myotubes. We found that mifepristone treatment alone in differentiated C2C12 myotubes significantly reduced *Klf15* levels at all three doses used when compared to untreated cells (Fig. 1B). Interestingly, mifepristone, whether added before or after dexamethasone, did not reduce *Klf15* expression in C2C12 myotubes when compared to dexamethasone-treated cells (Fig. 1C). Finally, the highest dose of mifepristone (10 μM) did not significantly impact cell death of C2C12 myotubes (Fig. 1D) or proliferation of C2C12 myoblasts (Fig. 1E) as compared to untreated cells, suggesting that although mifepristone treatment was safe, it did not reduce *Klf15* expression in GC-treated C2C12 myotubes.

In 3T3-L1 cells, there was no significant difference in *GRα* or *GRβ* expression between pre-adipocytes and differentiated adipocytes (Fig. 1F). Mifepristone treatment alone had no effect on *Klf15* levels in differentiated 3T3-L1 adipocytes when compared to untreated cells (Fig. 1G). Interestingly, all three doses of mifepristone significantly reduced *Klf15* expression when administered after dexamethasone in differentiated 3T3-L1 adipocytes when compared to dexamethasone treatment alone (Fig. 1H), suggesting that mifepristone can reduce *Klf15* levels in 3T3-L1 adipocytes only when *Klf15* expression is in a hyperactivated state. We found that the highest dose of mifepristone (10 μM) did not significantly impact cell death of 3T3-L1 adipocytes when compared to untreated cells (Fig. 1I). However, we did observe that the highest dose of mifepristone (10 μM) significantly reduced the proliferation of 3T3-L1 pre-adipocytes when compared to untreated cells (Fig. 1J).

As FL83B hepatocyte cells are already in a differentiated state, we directly compared expression of GR isoforms and found that the levels of *GRβ* are significantly lower than *GR*α (Fig. 1K). Mifepristone treatment alone in FL83B hepatocyte cells had no effect on *Klf15* expression (Fig. 1L). However, mifepristone significantly reduced *Klf15* expression at the higher doses of 5 μM and 10 μM concentrations after dexamethasone treatment when compared to dexamethasone alone (Fig. 1M). The highest dose of mifepristone (10 μM) applied prior to dexamethasone also significantly reduced *Klf15* expression (Fig. 1M). Similar to 3T3-L1 cells, we found that the highest dose of mifepristone (10 μM) did not significantly impact cell death of FL83B hepatocyte cells (Fig. 1N) but did significantly reduce proliferation of FL83B cells at the highest dose (Fig. 1O).

Combined, our in vitro experiments suggest that mifepristone’s effect on *Klf15* expression, cell viability and proliferation differs between metabolic tissue cell types.

### Mifepristone treatment ameliorates neuromuscular pathology in a severe *C. elegans* model of SMA

Next, we investigated the therapeutic potential of mifepristone in a validated severe SMA *C. elegans* invertebrate model^36,37^. The *C. elegans* nematode maintains functional conservation of neuronal processes and has high homology with the human genome^38^. *C. elegans* have a single *smn-1* gene that when diminished causes larval lethality, slowed growth and impaired neuromuscular function in pharyngeal pumping and mobility^39^.

We firstly set-out to assess the baseline expression of *Klf15* in SMA *C. elegans smn-1* (ok355) with the caveat that the *C. elegans* genome encodes only three Krüppel- like factors (1, 2 and 3)^40^ compared to 17 in mammals^41^. Furthermore, while the C2H2 zinc fingers are highly conserved between the two species, Klf-1, -2 and -3 in *C. elegans* are not direct orthologs of the mammalian Klf15^42^. Nevertheless, these analyses revealed that the expression of *Klf-1* and *Klf-3* is unchanged in SMA *C. elegans smn-1* (ok355) when compared to WT *C. elegans*, while the levels of *Klf-2* are significantly downregulated (Supplementary Fig. 2A).

**Fig. 2:**
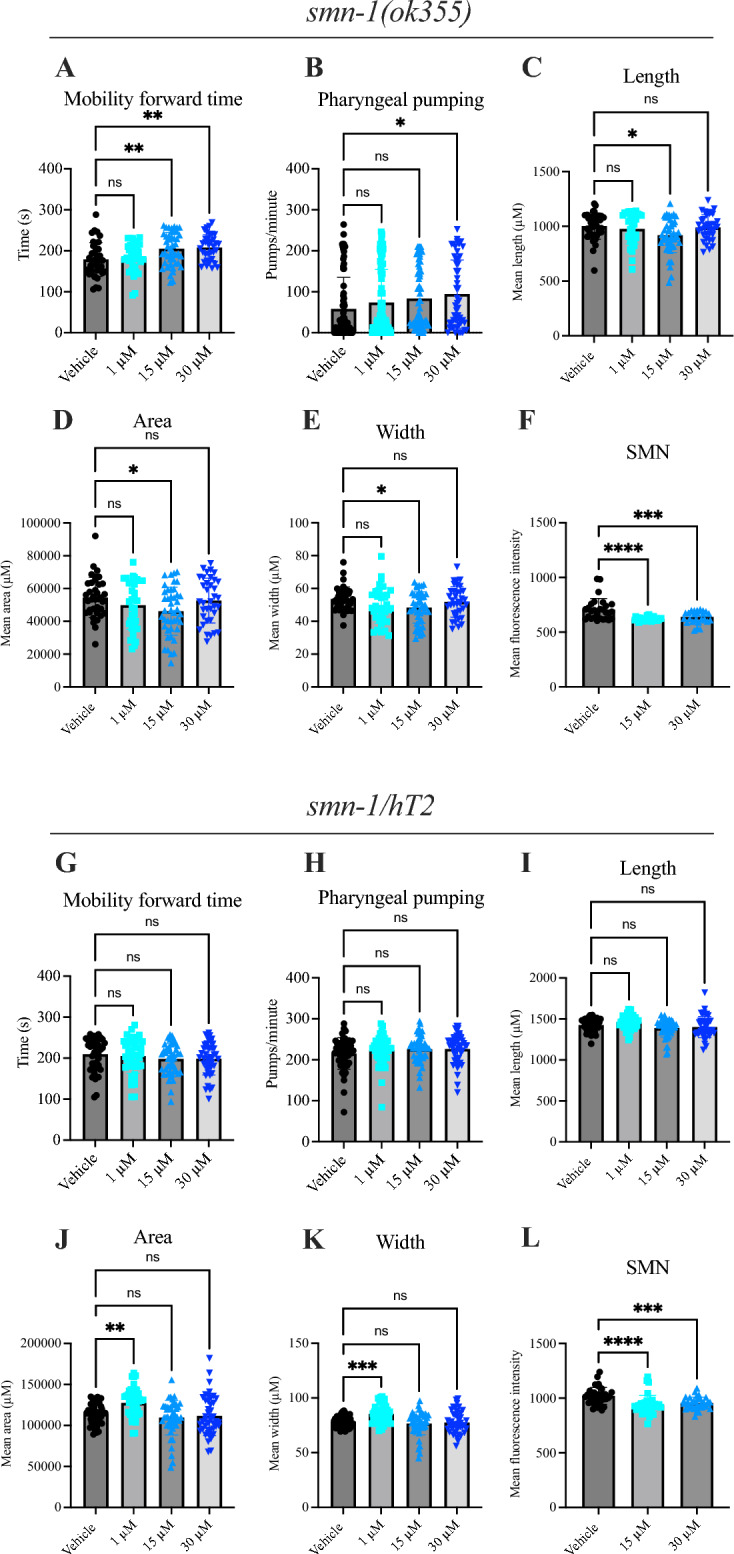
Mifepristone ameliorates the neuromuscular phenotype in a severe SMA *C. elegans smn-1* (ok355) model. (**A**) Mobility forward time filmed at 15 frames/second for 5 min in vehicle- or mifepristone-treated (1, 15 or 30 μM) SMA *C. elegans smn-1* (ok355). Data are mean ± SEM, N = 25 animals per experimental group, one-way ANOVA, ns = not significant, **P < 0.01. (**B**) Pharyngeal pumping rates (pumps/minute) defined as grinder movements in any axis at 175 frames/10 s in vehicle- or mifepristone-treated (1, 15 or 30 μM) SMA *C. elegans smn-1* (ok355). Data are mean ± SEM, N = 25 animals per experimental group, one-way ANOVA, ns = not significant, *P < 0.05. (**C**) Mean length in vehicle- or mifepristone-treated (1, 15 or 30 μM) SMA *C. elegans smn-1* (ok355). Data are mean ± SEM, N = 25 animals per experimental group, one-way ANOVA, ns = not significant, *P < 0.05. (**D**) Mean area in vehicle- or mifepristone-treated (1, 15 or 30 μM) SMA *C. elegans smn-1* (ok355). Data are mean ± SEM, N = 25 animals per experimental group, one-way ANOVA, ns = not significant, *P < 0.05. (**E**) Mean width in vehicle- or mifepristone-treated (1, 15 or 30 μM) SMA *C. elegans smn-1* (ok355). Data are mean ± SEM, N = 25 animals per experimental group, one-way ANOVA, ns = not significant, *P < 0.05. (**F**) SMN expression in vehicle- or mifepristone-treated (15 or 30 μM) SMA *C. elegans smn-1* (ok355). Data are mean ± SEM, N = 25 animals per experimental group, one-way ANOVA, ns = not significant, ***P < 0.001. ****0.0001. (**G**) Mobility forward time filmed at 15 frames/ second for 5 min in vehicle- or mifepristone-treated (1, 15 or 30 μM) control *C. elegans smn-1/hT2*. Data are mean ± SEM, N = 25 animals per experimental group, one-way ANOVA, ns = not significant. (**H**) Pharyngeal pumping rates (pumps/minute) defined as grinder movements in any axis at 175 frames/10 s in vehicle- or mifepristone-treated (1, 15 or 30 μM) control *C. elegans smn-1/hT2*. One-way ANOVA was performed. Data are mean ± SEM, N = 25 animals per experimental group, one-way ANOVA, ns = not significant. (**I**) Mean length in vehicle- or mifepristone-treated (1, 15 or 30 μM) control *C. elegans smn-1/hT2*. Data are mean ± SEM, N = 25 animals per experimental group, one-way ANOVA, ns = not significant. (**J**) Mean area in vehicle- or mifepristone-treated (1, 15 or 30 μM) control *C. elegans smn-1/hT2*. Data are mean ± SEM, N = 25 animals per experimental group, one-way ANOVA, ns = not significant, **P < 0.01. (**K**) Mean width in vehicle- or mifepristone-treated (1, 15 or 30 μM) control *C. elegans smn-1/hT2*. Data are mean ± SEM, N = 25 animals per experimental group, one-way ANOVA, ns = not significant, ***P < 0.001. (**L**) SMN expression in vehicle- or mifepristone-treated (15 or 30 μM) control *C. elegans smn-1/hT2*. Data are mean ± SEM, N = 25 animals per experimental group, one-way ANOVA, ns = not significant, ***P < 0.001. ****0.0001.

Next, SMA *C. elegans smn-1* (ok355) and control *C. elegans smn-1/hT2* nematodes were treated with increasing doses of mifepristone (1, 15 and 30 μM) and compared to vehicle-treated controls. Interestingly, we observed that the higher 15 and 30 μM concentrations of mifepristone significantly increased mobility forward time in *C. elegans smn-1* (ok355) compared to vehicle-treated *C. elegans smn-1* (ok355) (Fig. 2A). Additionally, the highest dose of mifepristone (30 μM) significantly increased pharyngeal pumping in the *C. elegans smn-1* (ok355) model compared to vehicle-treated *C. elegans smn-1* (ok355) (Fig. 2B). In light of these improved motor function parameters, we also measured worm size as *smn-1* (ok355) worms are smaller in size compared to controls. We found that the length (Fig. 2C), area (Fig. 2D) and width (Fig. 2E) of *C. elegans smn-1* (ok355) were significantly reduced following 15 μM mifepristone when compared to vehicle-treated *C. elegans smn-1* (ok355), suggesting that mifepristone does not rescue the growth defects present in SMA worms. In contrast, survival (Supplementary Fig. 2B), distance travelled (Supplementary Fig. 2C), speed (Supplementary Fig. 2D) and reversal times (Supplementary Fig. 2E) remained unchanged in mifepristone-treated SMA *C. elegans smn-1* (ok355). Surprisingly, we observed a small but significant decrease in Smn levels in *C. elegans smn-1* (ok355) following treatment with 15 and 30 μM mifepristone (Fig. 2F), which does not appear to have overt detrimental effects based our other behavioural and functional assessments.

Interestingly, mifepristone did not impact mobility forward time (Fig. 2G), pharyngeal pumping (Fig. 2H) or length (Fig. 2I) in the *C. elegans smn-1/hT2* controls, while area (Fig. 2J) and width (Fig. 2K) were significantly increased following 1 μM mifepristone, suggesting a disease-specific effect of mifepristone on neuromuscular function in nematodes. Survival (Supplementary Fig. 2F), distance travelled (Supplementary Fig. 2G), speed (Supplementary Fig. 2H) and reversal times (Supplementary Fig. 2I) in control *C. elegans smn-1/hT2* nematodes were not impacted by mifepristone treatment. Finally, we also observed a small but significant decreased expression of *Smn* in mifepristone-treated *C. elegans smn-1/hT2* controls (Fig. 2 L), with no accompanying observable adverse effects.

Our experiments in a *C. elegans* SMA model shows that mifepristone treatment leads to some neuromuscular benefits and no adverse effects, despite Klf signalling being differently regulated between nematodes and mammals.

### Mifepristone treatment improves righting reflex and survival in *Smn*^*2B/-*^ SMA mice

**Fig. 3:**
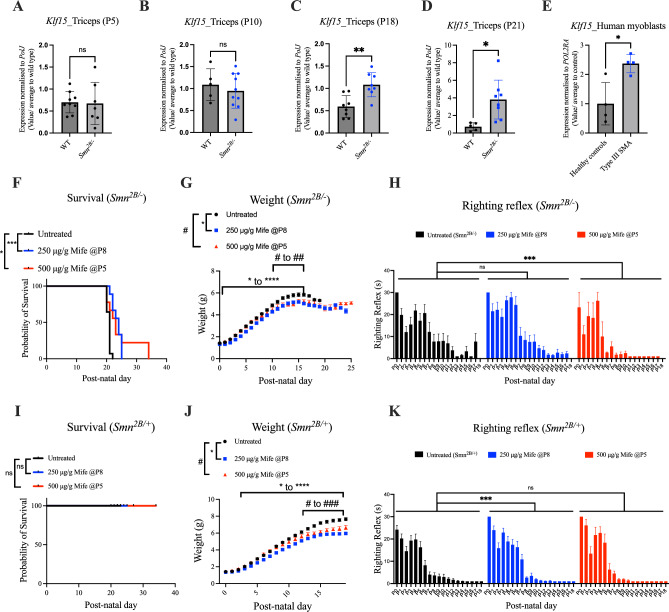
Mifepristone treatment improves survival of *Smn*^*2B/-*^ mice. (**A**) *Klf15* expression in triceps of post-natal day (P) 5 wild type (WT) and *Smn*^*2B/-*^ mice. Data are mean ± SEM, N = 5–8 animals per experimental group, unpaired *t-test*, ns = not significant. (**B**) *Klf15* expression in triceps of P10 WT and *Smn*^*2B/-*^ mice. Data are mean ± SEM, N = 5–8 animals per experimental group, unpaired *t-test*, ns = not significant. (**C**) *Klf15* expression in triceps of P18 WT and *Smn*^*2B/-*^ mice. Data are mean ± SEM, N = 5–8 animals per experimental group, unpaired *t-test*, **P < 0.001. (**D**) *Klf15* expression in triceps of post-natal day (P) 21 WT and *Smn*^*2B/-*^ mice. Data are mean ± SEM, N = 5–8 animals per experimental group, unpaired *t-test*, *P < 0.05. (**E**) *Klf15* expression in human control and SMA deltoid myoblasts, N = 4, unpaired *t-test*, *P < 0.05. (**F**) Survival curves of untreated and mifepristone-treated (250 μg/g starting at P8 or 500 μg/g starting at P5) *Smn*^*2B/-*^ mice. Data are Kaplan–Meier survival curves, N = 9–14 animals per experimental group, Log-rank (Mantel-Cox) test, *P < 0.05, ***P < 0.001. Survival data for selected optimal doses are repeated in supplemental Fig. 2A. (**G**) Daily weights of untreated and mifepristone-treated (250 μg/g starting at P8 or 500 μg/g starting at P5) *Smn*^*2B/-*^ mice. Data are mean ± SEM, N = 9–14 animals per experimental group, two-way ANOVA, */#P < 0.05, **/##P < 0.01, ****P < *0.0001.* (**H**) Daily righting reflex of untreated and mifepristone-treated (250 μg/g starting at P8 or 500 μg/g starting at P5) *Smn*^*2B/-*^ mice. Data are mean ± SEM, N = 9–14 animals per experimental group, one-way ANOVA, ns = not significant, ***P < 0.001. (**I**) Survival curves of untreated and mifepristone-treated (250 μg/g starting at P8 or 500 μg/g starting at P5) *Smn*^*2B/*+^ mice. Data are Kaplan–Meier survival curves, N = 11–14 animals per experimental group, Log-rank (Mantel-Cox) test, ns = not significant. (**J**) Daily weights of untreated and mifepristone-treated (250 μg/g starting at P8 or 500 μg/g starting at P5) *Smn*^*2B/*+^ mice. Data are mean ± SEM, N = 11–14 animals per experimental group, two-way ANOVA, */#P < 0.05, **/##P < 0.01, ***/###P < 0.001, ****P < *0.0001.* (**K**) Daily righting reflex of untreated and mifepristone-treated (250 μg/g starting at P8 or 500 μg/g starting at P5) *Smn*^*2B/*+^mice. Data are mean ± SEM, N = 11–14 animals per experimental group, one-way ANOVA, ns = not significant, ***P < 0.001.

Having evaluated the activity of mifepristone in relevant cell types and a *C. elegans* model of SMA, we next wanted to assess its therapeutic potential in a mammalian model, the *Smn*^*2B/-*^ intermediate SMA mice^43^. We first assessed the expression of *Klf15* in triceps of pre-symptomatic (post-natal day (P) 5), early symptomatic (P10), symptomatic (P18) and late symptomatic (P21) *Smn*^*2B/-*^ SMA mice and showed a significantly increased expression at P18 and P21 when compared to age-matched wild type (WT) mice (Fig. 3A–D), reproducing our previously published results where an increase in *Klf15* expression was seen after P11^3^. Using human primary myoblasts isolated from deltoid biopsies, we also observed significantly elevated levels of *Klf15* in myoblasts from SMA Type III patient samples compared to healthy controls (Fig. 3E), again supporting our previous study reporting elevated *Klf15* expression in human SMA muscle^3^. Both our past and current work therefore support the rationale behind reducing *Klf15* activity in SMA.

We initially conducted pilot studies to optimise the dosing regimen of mifepristone in *Smn*^*2B/-*^ mice, which determined that the best dosing regimens were 500 μg/g daily gavage starting at P5 and 250 μg/g daily gavage starting at P8 (Supplementary Fig. 3A) (median survival 21 days for untreated *Smn*^*2B/-*^ mice, 20 days for 125 μg/g-P5-treated *Smn*^*2B/-*^ mice, 20.5 days for 200 μg/g-P5-treated *Smn*^*2B/-*^ mice, 22 days for 250 μg/g-P5-treated *Smn*^*2B/-*^ mice, 24 days for 250 μg/g-P8-treated *Smn*^*2B/-*^ mice, 23 days for 500 μg/g-P5-treated *Smn*^*2B/-*^ mice, 21.5 days for 500 μg/g-P8-treated *Smn*^*2B/-*^ mice, 20 days for 1000 μg/g-P5-treated *Smn*^*2B/-*^ mice). We also found that the vehicle (0.5% carboxymethylcelluose (CMC)) did not affect weight, righting reflex or survival of *Smn*^*2B/-*^ SMA mice and *Smn*^*2B*/+^ healthy littermates when compared to untreated animals (Supplementary Fig. 3B–G).

As demonstrated in the pilot studies, both optimal mifepristone dosing regimens (500 μg/g daily gavage starting at P5 and 250 μg/g daily gavage starting at P8) significantly increased the lifespan of *Smn*^*2B/-*^ SMA mice compared to untreated *Smn*^*2B/-*^ animals (Fig. 3F) (median survival 21 days for untreated *Smn*^*2B/-*^ mice, 24 days for 250 μg/g-treated *Smn*^*2B/-*^ mice, 23 days for 500 μg/g-treated *Smn*^*2B/-*^ mice). In contrast, the weights of *Smn*^*2B/-*^ SMA mice were significantly decreased across both mifepristone-treated compared to untreated *Smn*^*2B/-*^ mice (Fig. 3G). Of note, this reduced weight occurred prior to treatment (P8) in the 250 μg/g experimental group, suggesting that this may be due to smaller weights at birth in those treated litters. We also saw a significant decrease in the time it took 500 μg/g mifepristone-treated *Smn*^*2B/-*^ mice to right themselves during disease progression compared to untreated *Smn*^*2B/-*^ mice (Fig. 3H). However, there was no significant difference in the righting reflex between 250 μg/g mifepristone-treated and untreated animals (Fig. 3H), suggesting that timing and dose of mifepristone lead to differential effects in *Smn*^*2B/-*^ mice. The lifespan of *Smn*^*2B/*+^ healthy control mice was not negatively affected by mifepristone (Fig. 3I). However, similar to *Smn*^*2B/-*^ mice, both dosing regimens of mifepristone significantly decreased the weight of *Smn*^*2B/*+^ animals (Fig. 3J), which preceded the first dose (P8) in 250 μg/g mifepristone-treated litters, further supporting intrinsic smaller weights at birth in that experimental cohort. Interestingly, the time to right in *Smn*^*2B/*+^ healthy littermates was significantly decreased following 250 μg/g mifepristone treatment, while there was no impact on righting reflex in 500 μg/g mifepristone-treated *Smn*^*2B/*+^ mice when compared to untreated *Smn*^*2B/*+^ animals (Fig. 3K). As 250 μg/g mifepristone was administered at a later time point, this could suggest that mifepristone’s effect in healthy control mice is treatment stage dependent.

Our data therefore support the beneficial effects of mifepristone in two different models of SMA, *C. elegans* and *Smn*^*2B/-*^ mice.

### Mifepristone significantly downregulates the expression of *Klf15* in BAT and increases myofiber area in skeletal muscle of *Smn*^*2B/-*^ mice

Next, we wanted to investigate the effect of mifepristone at molecular and histological levels in metabolically active skeletal muscle, liver and BAT of *Smn*^*2B/-*^ SMA and *Smn*^*2B/*+^ control mice. Of note, while *Smn*^*2B/*+^ mice are healthy littermates in terms of lifespan and reproduction, they do have reduced levels of Smn, which has been shown to impact gene expression and metabolism^44^. To limit confounding variables, we made comparisons between untreated and treated *Smn*^*2B/-*^ mice to determine if the effects were SMA dependent or independent.

**Fig. 4:**
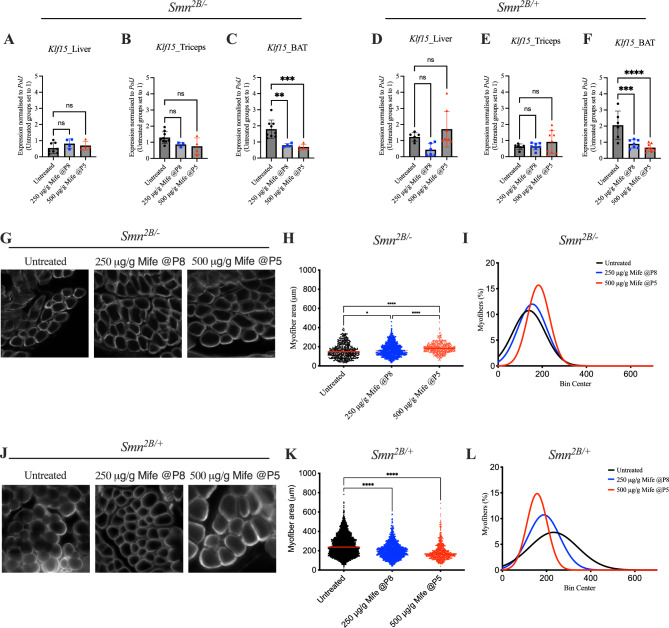
Mifepristone impacts disease phenotypes in *Smn*^*2B/-*^ mice in a tissue and disease-state specific manner. (**A**) *Klf15* expression in liver from post-natal day (P) 18 untreated and mifepristone-treated (250 μg/g starting at P8 or 500 μg/g starting at P5) *Smn*^*2B/-*^ mice. Data are mean ± SEM, N = 4–8 animals per experimental group, one-way ANOVA, ns = not significant. (**B**) *Klf15* expression in triceps from P18 untreated and mifepristone-treated (250 μg/g starting at P8 or 500 μg/g starting at P5) *Smn*^*2B/-*^ mice. Data are mean ± SEM, N = 3–8 animals per experimental group, one-way ANOVA, ns = not significant. (**C**) *Klf15* expression in brown adipose tissue (BAT) from P18 untreated and mifepristone-treated (250 μg/g starting at P8 or 500 μg/g starting at P5) *Smn*^*2B/-*^ mice. Data are mean ± SEM, N = 4–8 animals per experimental group, one-way ANOVA, **P < 0.01, ***P < 0.001. (**D**) *Klf15* expression in liver from P18 untreated and mifepristone-treated (250 μg/g starting at P8 or 500 μg/g starting at P5) *Smn*^*2B/*+^ mice. Data are mean ± SEM, N = 6–8 animals per experimental group, one-way ANOVA, ns = not significant. (**E**) *Klf15* expression in triceps from P18 untreated and mifepristone-treated (250 μg/g starting at P8 or 500 μg/g starting at P5) *Smn*^*2B/*+^ mice. Data are mean ± SEM, N = 6–9 animals per experimental group, one-way ANOVA, ns = not significant. (**F**) *Klf15* expression in BAT from P18 untreated and mifepristone-treated (250 μg/g starting at P8 or 500 μg/g starting at P5) *Smn*^*2B/*+^ mice. Data are mean ± SEM, N = 6–9 animals per experimental group, one-way ANOVA, ***P < 0.001, ****P < 0.0001. (**G**) Representative images of laminin-stained cross-sections of tibialis anterior (TA) muscles from P18 untreated and mifepristone-treated (250 μg/g starting at P8 or 500 μg/g starting at P5) *Smn*^*2B/-*^ mice. (**H**) Quantification of myofiber area of laminin-stained cross-sections of TA muscles from P18 untreated and mifepristone-treated (250 μg/g starting at P8 or 500 μg/g starting at P5) *Smn*^*2B/-*^ mice. Data are dot plot and mean, n = 3–4 animals per experimental group (> 200 myofibers per experimental group), one-way ANOVA, *P < 0.05, ****P < 0.0001. (**I**) Relative frequency distribution of myofiber size in TA muscles from P18 untreated and mifepristone-treated (250 μg/g starting at P8 or 500 μg/g starting at P5) *Smn*^*2B/-*^ mice. (**J**) Representative images of laminin-stained cross-sections of TA muscles from P18 untreated and mifepristone-treated (250 μg/g starting at P8 or 500 μg/g starting at P5) *Smn*^*2B/*+^ mice. (**K**) Quantification of myofiber area of laminin-stained cross-sections of TA muscles from P18 untreated and mifepristone-treated (250 μg/g starting at P8 or 500 μg/g starting at P5) *Smn*^*2B/*+^ mice. Data are dot plot and mean, n = 3–4 animals per experimental group (> 200 myofibers per experimental group), one-way ANOVA, ****P < 0.0001. (**L**) Relative frequency distribution of myofiber size in TA muscles from P18 untreated and mifepristone-treated (250 μg/g starting at P8 or 500 μg/g starting at P5) *Smn*^*2B/*+^ mice.

Firstly, we investigated *Klf15* expression in tissues from P18 untreated, 250 μg/g (P8) and 500 μg/g (P5) mifepristone-treated mice. In *Smn*^*2B/-*^ mice, we observed that *Klf15* expression in liver and triceps remained unchanged between untreated and mifepristone-treated *Smn*^*2B/-*^ animals (Fig. 4A-B). However, both doses of mifepristone significantly reduced *Klf15* expression in BAT of *Smn*^*2B/-*^ mice compared to untreated *Smn*^*2B/-*^ animals (Fig. 4C). Similar results were found in liver, triceps and BAT of *Smn*^*2B/*+^ mice (Fig. 4D–F), suggesting increased activity of the GR antagonist in adipose tissue, likely due to the increased metabolic rate of BAT.

As muscle atrophy is a canonical pathology of SMA, we next analysed myofiber area of the *tibialis anterior* (TA) muscle from P18 untreated, 250 μg/g and 500 μg/g mifepristone-treated *Smn*^*2B/-*^ and *Smn*^*2B/*+^ mice. Treatment with both doses of mifepristone significantly increased myofiber size in mifepristone-treated *Smn*^*2B/-*^ mice when compared to untreated *Smn*^*2B/-*^ animals (Fig. 4G-I). Interestingly, myofiber area in *Smn*^*2B/*+^ healthy littermate controls was significantly decreased following 250 μg/g and 500 μg/g mifepristone treatment when compared to untreated *Smn*^*2B/*+^ animals (Fig. 4J–L).

Finally, we assessed the effect of mifepristone on *Smn* expression and observed that even the highest dose of mifepristone (500 μg/g) had no effect on *Smn* levels in both *Smn*^*2B/-*^ and *Smn*^*2B/*+^ mice (Supplementary Fig. 4).

Overall, our molecular and histological analyses show tissue- and disease state-specific effects of mifepristone on skeletal muscle, liver and BAT.

### Mifepristone affects the expression of canonical metabolic effectors in *Smn*^*2B/-*^ mice

As mifepristone has been proposed as a potential treatment for metabolic syndrome^45^, we set out to determine if mifepristone improved previously reported metabolic perturbations in SMA.

**Fig. 5:**
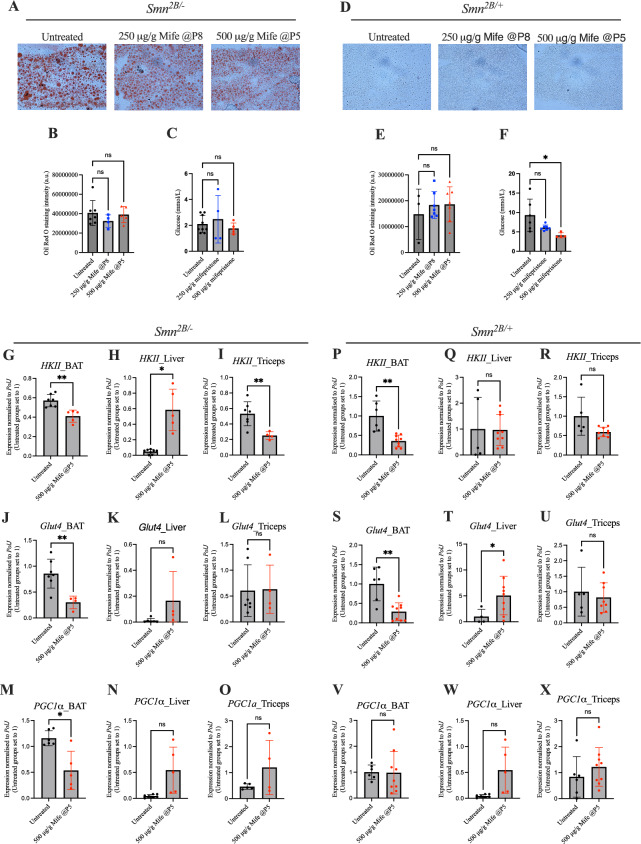
Mifepristone impacts metabolic markers in *Smn*^*2B/-*^ mice in a tissue and disease-state specific manner. (**A**) Representative images of oil-red-O-stained liver sections from post-natal day (P) 18 untreated and mifepristone-treated (250 μg/g starting at P8 or 500 μg/g starting at P5) *Smn*^*2B/-*^ mice. (**B**) Quantification of oil-red-O staining intensity in liver sections P18 untreated and mifepristone-treated (250 μg/g starting at P8 or 500 μg/g starting at P5) *Smn*^*2B/-*^ mice. Data are mean ± SEM, N = 4–7 animals per experimental group, one-way ANOVA, ns = not significant. (**C**) non-fasted glucose levels in P18 untreated, 250 μg/g and 500 μg/g mifepristone-treated *Smn*^*2B/-*^ mice. Data are mean ± SEM, N = 5–7 animals per experimental group, unpaired *t-test*, ns = not significant. (**D**) Representative images of oil-red-O-stained liver sections from P18 untreated and mifepristone-treated (250 μg/g starting at P8 or 500 μg/g starting at P5) *Smn*^*2B/*+^ mice. (**E**) Quantification of oil-red-O staining intensity in liver sections P18 untreated and mifepristone-treated (250 μg/g starting at P8 or 500 μg/g starting at P5) *Smn*^*2B/*+^ mice. Data are mean ± SEM, N = 3–7 animals per experimental group, one-way ANOVA, ns = not significant. (**F**) non-fasted glucose levels in P18 untreated, 250 μg/g and and 500 μg/g mifepristone-treated *Smn*^*2B/*+^ mice. Data are mean ± SEM, N = 5–7 animals per experimental group, unpaired *t-test*, *P < 0.05. (**G–I**)**,** *HKII* expression in BAT (**G**), liver (**H**) and triceps (**I**) from P18 untreated and 500 μg/g mifepristone-treated *Smn*^*2B/-*^ mice. Data are mean ± SEM, N = 5–7 animals per experimental group, unpaired *t-test*, *P < 0.05, **P < 0.01. (**J–L**)**,** *Glut4* expression in BAT (**J**), liver (**K**) and triceps (**L**) from P 18 untreated and 500 μg/g mifepristone-treated *Smn*^*2B/-*^ mice. Data are mean ± SEM, N = 5–7 animals per experimental group, unpaired *t-test*, **P < 0.01, ns = not significant. (**M–O**)**,** *PGC1α* expression in BAT (**M**), liver (**N**) and triceps (**O**) from P18 untreated and 500 μg/g mifepristone-treated *Smn*^*2B/-*^ mice. Data are mean ± SEM, N = 5–7 animals per experimental group, unpaired *t-test*, *P < 0.05, ns = not significant. (**P–R**)**,** *HKII* expression in BAT (**P**), liver (**Q**) and triceps (**R**) from P18 untreated and 500 μg/g mifepristone-treated *Smn*^*2B/*+^ mice. Data are mean ± SEM, N = 5–7 animals per experimental group, unpaired *t-test*, **P < 0.01. (**S–U**) *Glut4* expression in BAT (**S**), liver (**T**) and triceps (**U**) from P18 untreated and 500 μg/g mifepristone-treated *Smn*^*2B/*+^ mice. Data are mean ± SEM, N = 5–7 animals per experimental group, unpaired *t-test*, *P < 0.05, **P < 0.01, ns = not significant. (**V**) *PGC1α* expression in BAT (V), liver (W) and triceps (X) from P18 untreated and 500 μg/g mifepristone-treated *Smn*^*2B/*+^ mice. Data are mean ± SEM, N = 5–7 animals per experimental group, unpaired *t-test*, ns = not significant.

Mifepristone has been successfully used to treat fatty liver disease in patients with CS^46^, therefore, we used an oil-red-O stain to assess whether mifepristone affected the previously reported lipid accumulation in the liver of *Smn*^*2B/-*^ mice^10^. Quantification of oil-red-O staining intensity in livers from P18 animals showed no significant differences between untreated *Smn*^*2B/-*^ mice and mifepristone-treated *Smn*^*2B/-*^ animals (Fig. 5A,B). We also measured glucose levels in non-fasted P18 animals as we, and others, have previously observed glucose metabolism defects in SMA mice^13^. We found no significant differences in glucose levels between untreated and mifepristone-treated *Smn*^*2B/-*^ mice (Fig. 5C).

Similar results were observed in *Smn*^*2B/*+^ mice where there was a noticeable absence of hepatic lipid accumulation (Fig. 5D,E). Interestingly, glucose levels were significantly decreased following 500 μg/g mifepristone treatment in *Smn*^*2B/*+^ mice when compared to untreated control animals (Fig. 5F).

Finally, we investigated the expression of *hexokinase II* (*HKII*), *glucose transporter type 4* (*Glut4*) and *peroxisome proliferator-activated receptor-γ coactivator* (*PGC1α*), which we have previously demonstrated to be aberrantly expressed in SMA mice^44^. As oil-red-O and glucose levels analyses only revealed a significant change following the highest dose of mifepristone, we limited our comparisons between BAT, liver and triceps of P18 untreated and 500 μg/g mifepristone-treated mice.

In *Smn*^*2B/-*^ mice, we found that the *HKII* levels were significantly downregulated in BAT (Fig. 5G), upregulated in liver (Fig. 5H) and downregulated in triceps (Fig. 5I) of mifepristone-treated *Smn*^*2B/-*^ mice compared to untreated animals. *Glut4* expression was significantly decreased in BAT (Fig. 5J) and unchanged in liver (Fig. 5K) and triceps (Fig. 5L) of *Smn*^*2B/-*^ mice compared to untreated animals. Mifepristone had a similar impact on the expression of *PGC1α* in *Smn*^*2B/-*^ mice (Fig. 5M,N,O).

In control *Smn*^*2B/*+^ mice, *HKII* expression was significantly downregulated in mifepristone-treated animals (Fig. 5P), similar to what was observed in *Smn*^*2B/-*^ animals. In contrast, *HKII* levels remained unchanged in liver (Fig. 5Q) and triceps (Fig. 5R) of mifepristone-treated *Smn*^*2B/*+^ mice. Similar to *Smn*^*2B/-*^ mice, *Glut4* levels were significantly decreased in mifepristone-treated *Smn*^*2B/*+^ animals (Fig. 5S). In liver, *Glut4* expression was significantly increased (Fig. 5T), while it was unaffected in triceps (Fig. 5U) following mifepristone treatment. Finally, *PGC1α* expression remained unchanged in BAT, liver and triceps of mifepristone-treated *Smn*^*2B/*+^ mice (Fig. 5V,W,X).

Overall, our results show that mifepristone administration impacts a variety of metabolic markers in a dose- and disease-dependent manner.

### Mifepristone treatment is well tolerated but ineffective in the severe ‘Taiwanese’ SMA mouse model

As there is a wide range of SMA mouse models with differing disease onsets and progression, we next wanted to investigate mifepristone treatment in a more severe mouse model. We treated severe Taiwanese *Smn*^*-/-*^*;SMN2* mice^47^ with mifepristone daily starting at P3. An earlier start date (P3 vs P5 in *Smn*^*2B/-*^ mice) was selected due to the earlier and more severe disease onset in these mice. We found that there were no significant differences in survival (untreated and treated *Smn*^*2B/-*^ median survival 10 days respectively), weight and righting reflex between vehicle-treated and mifepristone-treated *Smn*^*-/-*^*;SMN2* mice compared to untreated animals (Supplementary Fig. 5A–C). In addition, no adverse effects were observed in mifepristone-treated *Smn*^+*/-*^*;SMN2* healthy littermates compared to untreated and vehicle-treated animals (Supplementary Fig. 5D–F). Overall, our results highlight key differences between SMA mouse models in their response to mifepristone, specifically demonstrating the more aggressive nature of phenotypes in the Taiwanese model making it much harder to rescue. This is in line with previous studies demonstrating differential therapeutic effects of interventions in different SMA mouse models^3,48^.

### Combinatorial treatment of scAAV9-*SMN1* with 500 μg/g mifepristone leads to selective synergistic effects in *Smn*^*2B/-*^ SMA mice

To address the benefit of combinatorial therapy for SMA, we combined daily administration of mifepristone (from P5 to P21) with an Onasemnogene abeparvovec-like vector (scAAV9-*SMN1*)^49^ given at P0 (day of birth) via facial intravenous injection (IV) with a scAAV9-*GFP* vector used as a control. Even though both doses of mifepristone improved survival of *Smn*^*2B/-*^ mice, 22% of mice survived an additional 10 days longer (to P35) following 500 μg/g compared to 250 μg/g mifepristone. Mifepristone (500 μg/g) produced a substantially greater improvement in motor function and myofiber hypertrophy than the alternative dose, without any adverse effects in healthy littermate controls. Furthermore, 500 μg/g mifepristone was administered at the earlier age of P5 (in contrast to 250 μg/g mifepristone (P8)), a translational time point likely to produce greater therapeutic outcome and correspond with administration to human patients with SMA. Taken together, we moved forward with 500 μg/g mifepristone to assess combinatorial therapy.

We assessed phenotypic outcomes, including weight and righting reflex in *Smn*^*2B/-*^ and *Smn*^*2B/*+^ mice. Daily weights were recorded from birth (P0) until P28 and weekly weights were recorded from P28 onwards. Of note, as scAAV9-*GFP*-injected *Smn*^*2B/-*^ SMA mice did not survive past weaning (P21), it was only possible to collect data from scAAV9-*SMN1* and scAAV9-*SMN1* + 500 μg/g mifepristone animals. Furthermore, none of the animals treated with scAAV9-*SMN1* died following treatment and were sacrificed for tissue harvesting purposes only.

In pre-weaned *Smn*^*2B/-*^ animals, we found no significant differences between scAAV9-*SMN1*-injected animals compared to scAAV9-*SMN1* + 500 μg/g mifepristone (Supplementary Fig. 6A). However, scAAV9-*GFP*-injected *Smn*^*2B/-*^ SMA mice weighed significantly less than scAAV9-*SMN1*-injected *Smn*^*2B/-*^ SMA mice and the scAAV9-*SMN1* + 500 μg/g mifepristone *Smn*^*2B/-*^ SMA mice as disease progressed, due to the lack of therapeutic activity of this control vector (Supplementary Fig. 6A). Interestingly, while the righting reflex of scAAV9-*SMN1*-injected *Smn*^*2B/-*^ animals was like those treated with scAAV9-*SMN1* + 500 μg/g mifepristone, there was a significant improvement of the righting reflex in scAAV9-*SMN1*-injected *Smn*^*2B/-*^ SMA mice compared to the control vector, scAAV9-*GFP*-injected *Smn*^*2B/-*^ SMA mice (Supplementary Fig. 6B). As weight differences become noticeable between the sexes post-weaning, we separated male and female mice from beyond this time-point. We observed no significant difference in weight between scAAV9-*SMN1*-injected *Smn*^*2B/-*^ males and females compared to males and females treated with scAAV9-*SMN1* + 500 μg/g mifepristone from P28 to humane endpoint (Supplementary Fig. 6C,D). Similar analyses in *Smn*^*2B/*+^ healthy control littermates revealed comparable weights and righting reflex between scAAV9-*GFP*-injected, scAAV9-*SMN1*-injected and those treated with scAAV9-*SMN1* + 500 μg/g mifepristone (Supplementary Fig. 6E–H).

**Fig. 6:**
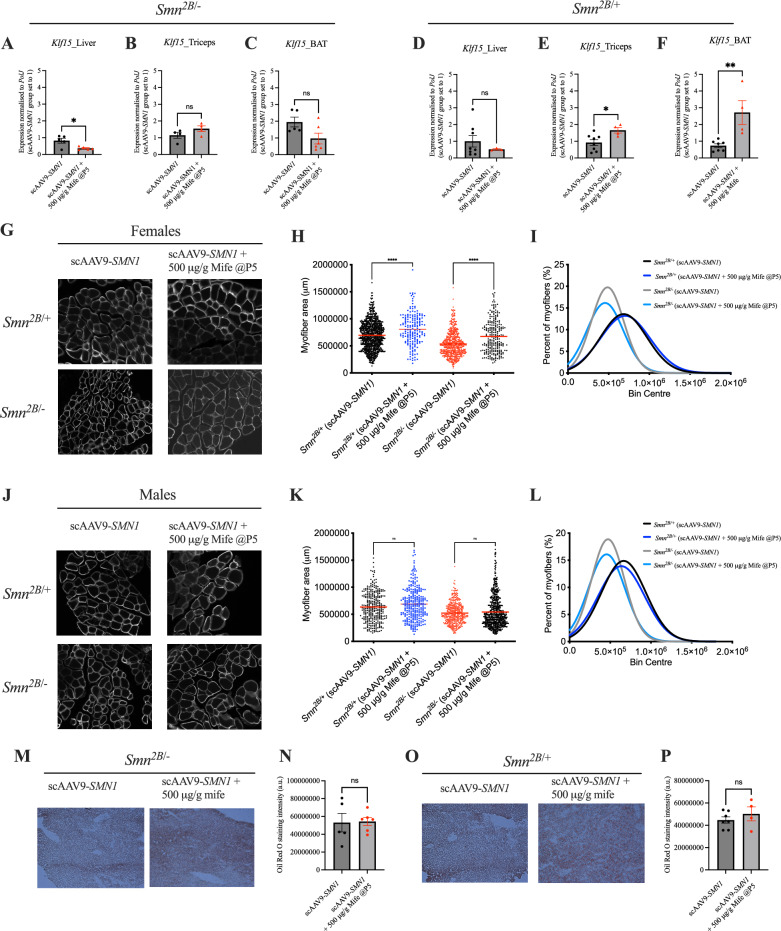
Combinatorial treatment of scAAV9-*SMN1* and mifepristone reduces *Klf15* expression in the liver of *Smn*^*2B/-*^ mice and significantly increases myofiber area in females specifically. (**A**), *Klf15* expression in liver from 6-month-old *Smn*^*2B/-*^ mice following treatment with scAAV9-*SMN1* at post-natal day (P) 0 or a combination of scAAV9-*SMN1* + mifepristone (500 μg/g from P5-P21). Data are mean ± SEM, N = 5 animals per experimental group, unpaired *t-test*, *P < 0.05. (**B**), *Klf15* expression in triceps from 6-month-old *Smn*^*2B/-*^ mice following treatment with scAAV9-*SMN1* at post-natal P0 or a combination of scAAV9-*SMN1* + mifepristone (500 μg/g from P5-P21). Data are mean ± SEM, N = 4 animals per experimental group, unpaired *t-test*, ns = not significant. (**C**), *Klf15* expression in brown adipose tissue (BAT) from 6-month-old *Smn*^*2B/-*^ mice following treatment with scAAV9-*SMN1* at post-natal day (P) 0 or a combination of scAAV9-*SMN1* + mifepristone (500 μg/g from P5-P21). Data are mean ± SEM, N = 5 animals per experimental group, unpaired *t-test*, ns = not significant. (**D**), *Klf15* expression in liver from 6-month-old *Smn*^*2B/*+^ mice following treatment with scAAV9-*SMN1* at post-natal P0 or a combination of scAAV9-*SMN1* + mifepristone (500 μg/g from P5-P21). Data are mean ± SEM, N = 4–8 animals per experimental group, unpaired *t-test*, ns = not significant. (**E**), *Klf15* expression in triceps from 6-month-old *Smn*^*2B/*+^ mice following treatment with scAAV9-*SMN1* at post-natal P0 or a combination of scAAV9-*SMN1* and mifepristone (500 μg/g from P5-P21). Data are mean ± SEM, N = 4–8 animals per experimental group, unpaired *t-test*, ns = not significant. (**F**), *Klf15* expression in BAT from 6-month-old *Smn*^*2B/*+^ mice following treatment with scAAV9-*SMN1* at post-natal P0 or a combination of scAAV9-*SMN1* + mifepristone (500 μg/g from P5-P21). Data are mean ± SEM, N = 4–8 animals per experimental group, unpaired *t-test*, **P < 0.01. (**G**), Representative images of laminin-stained cross-sections of *tibialis anterior* (TA) muscles from 6-month-old *Smn*^*2B/-*^ and *Smn*^*2B/*+^ females following treatment with scAAV9-*SMN1* at post-natal P0 or a combination of scAAV9-*SMN1* + mifepristone (500 μg/g from P5-P21). (**H**), Quantification of myofiber area of laminin-stained cross-sections of TA muscles from 6-month-old *Smn*^*2B/-*^ and *Smn*^*2B/*+^ females following treatment with scAAV9-*SMN1* at post-natal P0 or a combination of scAAV9-*SMN1* + mifepristone (500 μg/g from P5-P21). Data are dot plot and mean, n = 3–5 animals per experimental group (> 200 myofibers per experimental group), one-way ANOVA, ****P < 0.0001. (**I**), Relative frequency distribution of myofiber size in TA muscles from 6-month-old *Smn*^*2B/-*^ and *Smn*^*2B/*+^ females following treatment with scAAV9-*SMN1* at post-natal P0 or a combination of scAAV9-*SMN1* + mifepristone (500 μg/g from P5-P21). (**J**), Representative images of laminin-stained cross-sections of TA muscles from 6-month-old *Smn*^*2B/-*^ and *Smn*^*2B/*+^ males following treatment with scAAV9-*SMN1* at post-natal P0 or a combination of scAAV9-*SMN1* + mifepristone (500 μg/g from P5-P21). (**K**), Quantification of myofiber area of laminin-stained cross-sections of TA muscles from 6-month-old *Smn*^*2B/-*^ and *Smn*^*2B/*+^ males following treatment with scAAV9-*SMN1* at post-natal P0 or a combination of scAAV9-*SMN1* and mifepristone (500 μg/g from P5-P21). Data are dot plot and mean, n = 3–5 animals per experimental group (> 200 myofibers per experimental group), one-way ANOVA, ns = not significant. (**L**), Relative frequency distribution of myofiber size in TA muscles from 6-month-old *Smn*^*2B/-*^ and *Smn*^*2B/*+^ males following treatment with scAAV9-*SMN1* at post-natal P0 or a combination of scAAV9-*SMN1* + mifepristone (500 μg/g from P5-P21). (**M**), Representative images of oil red O-stained liver sections from 6-month-old *Smn*^*2B/-*^ mice following treatment with scAAV9-*SMN1* at post-natal P0 or a combination of scAAV9-*SMN1* + mifepristone (500 μg/g from P5-P21). (**N**), Quantification of oil red O staining intensity in liver sections from 6-month-old *Smn*^*2B/-*^ mice following treatment with scAAV9-*SMN1* at post-natal P0 or a combination of scAAV9-*SMN1* + mifepristone (500 μg/g from P5-P21). Data are mean ± SEM, N = 5–6 animals per experimental group, unpaired *t-test*, ns = not significant. (**O**), Representative images of oil red O-stained liver sections from 6-month-old *Smn*^*2B/*+^ mice following treatment with scAAV9-*SMN1* at post-natal P0 or a combination of scAAV9-*SMN1* + mifepristone (500 μg/g from P5-P21). (**P**), Quantification of oil red O staining intensity in liver sections from 6-month-old *Smn*^*2B/*+^ mice following treatment with scAAV9-*SMN1* at post-natal P0 or a combination of scAAV9-*SMN1* + mifepristone (500 μg/g from P5-P21). Data are mean ± SEM, N = 4–6 animals per experimental group, unpaired *t-test*, ns = not significant.

We also undertook molecular and histological investigations in tissues from 6-month-old *Smn*^*2B/-*^ SMA mice and *Smn*^*2B/*+^ control mice that either received a single injection of scAAV9-*SMN1* or were treated with the combinatorial intervention of scAAV9-*SMN1* + 500 μg/g mifepristone. We assessed *Klf15* expression in liver, skeletal muscle (triceps) and BAT. In *Smn*^*2B/-*^ SMA mice *Klf15* expression was significantly reduced in the liver (Fig. 6A), while remaining unchanged in the triceps (Fig. 6B) and BAT (Fig. 6C) of animals treated with scAAV9-*SMN1* + 500 μg/g mifepristone compared to scAAV9-*SMN1* alone. In *Smn*^*2B/*+^ control mice, *Klf15* expression in liver (Fig. 6D) remained unchanged in animals treated with scAAV9-*SMN1* + 500 μg/g mifepristone compared to scAAV9-*SMN1* alone, whereas there was a significant increase of *Klf15* expression in triceps and BAT following scAAV9-*SMN1* + 500 μg/g mifepristone when compared to scAAV9-*SMN1* alone (Fig. 6E-F). Our results therefore suggest disease state and tissue-specific effect the combinatorial intervention on *Klf15* expression.

Next, we assessed myofiber area, whereby the analyses were separated by sex. We observed that the combination treatment of scAAV9-*SMN1* + 500 μg/g mifepristone significantly increased the myofiber area of both *Smn*^*2B/-*^ SMA mice and *Smn*^*2B/*+^ control females compared to scAAV9-*SMN1* alone (Fig. 6G-I). In contrast, there were not significant differences in the myofiber area between treatment groups in males (Fig. 6J–L), suggesting a sex-dependent effect of the combinatorial approach with mifepristone as an adjunct therapy.

Finally, we investigated whether scAAV9-*SMN1* + 500 μg/g mifepristone impacted lipid accumulation in the liver, as assessed via oil-red-O staining intensity. Lipid accumulation remained unchanged in the liver of both *Smn*^*2B/-*^ SMA mice and *Smn*^*2B/*+^ control mice treated with either scAAV9-*SMN1* or scAAV9-*SMN1* + 500 μg/g mifepristone (Fig. 6M–P), supporting previous research demonstrating rescue of the fatty liver phenotype by scAAV9-*SMN1* therapy^49^.

## Discussion

In this study, we addressed two key research questions. Firstly, whether targeting the GC-GR-Klf15 metabolic pathway via mifepristone could improve disease phenotypes in SMA animal models^3^. Secondly, whether combining an *SMN1*-directed gene therapy with mifepristone could lead to additional benefits in peripheral and metabolic tissues compared to the gene therapy alone. Overall, we found that repurposing mifepristone for the treatment of SMA reduced *Klf15* expression specifically in BAT, increased the lifespan and muscle size of treated *Smn*^*2B/-*^ mice, and improved motor function in a SMA *C. elegans* model. Furthermore, combining mifepristone with an scAAV9-*SMN1* gene therapy resulted in tissue-, sex and disease state-specific improved pathological changes compared to the gene therapy alone.

In the first part of this study, we evaluated mifepristone activity in cell models of peripheral tissues. Across all three models (C2C12 (muscle), 3T3-L1 (adipose) and FL83B (liver)), treatment with mifepristone reduced dexamethasone-induced *Klf15* expression in a cell and differentiation state-dependent manner. Key players in mifepristone’s mode of action are the isoforms of the GR (α and β), whereby GRα is the main mediator of GCs while GRβ inhibits GRα and induces GC resistance^34,35^. We found that expression of both GR isoforms (α and β) were increased in untreated C2C12 myotubes compared to C2C12 myoblasts, suggesting that the concomitant upregulation of GRβ in myotubes may counteract the aberrant activation of GRα. Previous research overexpressing GRβ (GRβOE) in C2C12 cells showed that GC-induced leucine zipper (GILZ), a target of GRα, was significantly reduced in GRβOE myoblasts, demonstrating a reduction in GRα activity when GRβ expression is elevated^48^. This could explain the inability of mifepristone to antagonise GRα and reduce dexamethasone-induced *Klf15* expression in our treated C2C12 myotubes. Mifepristone’s inhibition of *Klf15* expression may therefore work better when given early during muscle development.

Interestingly, in FL83B hepatocyte-like cells, mifepristone reduced *Klf15* expression regardless of dexamethasone treatment, which might be explained by the observation that FL83B cells displayed higher levels of *GRα* than *GRβ* in a differentiated state. Lower levels of GRβ suggests that mifepristone is able to actively bind GRα and antagonise transcriptional regulation without resistance from GRβ’s negative regulation^50^. In fact, previous research has shown that small GR antagonist molecules that are active in the liver can successfully ameliorate metabolic syndrome in rats^51^, suggesting that modulation of the GR pathway could help reduce known metabolic liver pathology in SMA^12,19^.

Although our study’s limitation is that we did not directly compare *Klf15* levels in the liver and BAT of treated *Smn*^*2B/-*^ mice with those of *Smn*^*2B/*+^ or WT control mice, and additional experiments should be planned to confirm, a key finding from our molecular analyses of mifepristone-treated tissues is the selective downregulation of *Klf15* expression in BAT of both *Smn*^*2B/-*^ SMA mice and *Smn*^*2B/*+^ healthy littermate controls. The previously reported increased expression of *Klf15* in BAT of SMA mice^3^ may disrupt *Klf15*’s ability to maintain crucial processes such as lipid metabolism. Specifically, *Klf15* has been demonstrated to regulate fuel switching between glucose and fatty acids in response to changes in energy status in BAT^52^. The imbalance of *Klf15* levels in BAT of SMA mice may therefore contribute to aberrant gluconeogenesis and insulin resistance as a result of reduced metabolic flexibility^52^. Reducing *Klf15* expression in BAT with mifepristone may potentially improve metabolic homeostasis in that tissue. Additionally, cross-talk occurs between BAT and pancreatic cells in obese mice, whereby the overexpression of *Klf15* in BAT from obese mice enhanced insulin secretion from pancreatic β-cells^53^. As previous studies have reported a particular reduction of pancreatic β-cells in *Smn*^*2B/-*^ mice^13^, we speculate that the increased expression of *Klf15* seen in BAT of SMA mice could be a compensatory mechanism due to the reduced activity of pancreatic β-cells^13^. Mifepristone’s ability to reduce *Klf15* in BAT of *Smn*^*2B/-*^ mice could thus improve insulin resistance and metabolic pathologies through crosstalk between adipose and pancreatic tissues^54^. While inhibition of said compensatory mechanism may seem counterintuitive, mifepristone treatment did not impact glucose levels in non-fasted *Smn*^*2B/-*^ mice. Nevertheless, as it is most likely that SMN-independent therapies will be administered alongside an already approved disease modifying therapy that most likely restores glucose metabolism homeostasis, the potential benefits of mifepristone and other similar approaches on additional metabolic pathologies warrants their exploration. Furthermore, to address if mifepristone affected other metabolic perturbations, we looked at the expression of metabolic genes previously reported as being impacted in SMA (*HKII*, *Glut4* and *PGC1α*)^3,44^. We found that mifepristone impacted their expression in a tissue- and disease state-dependent manner, suggesting possible activity on glycolysis, mitochondrial function and glucose metabolism^55–61^.

Our in vivo studies also revealed a significant increase in myofiber area of *Smn*^*2B/-*^ SMA mice following mifepristone treatment. This increase in myofiber size is likely associated with the improved righting reflex times seen after mifepristone treatment (specifically 500 μg/g at P5) in *Smn*^*2B/-*^ mice. Klf15 plays a role in muscle physiology and exercise adaptation by regulating lipid flux, which could explain our current findings^45^. Furthermore, the knockdown of DDIT4 (REDD1), another GC regulated protein, boosts muscle mass supporting the concept that targeting GC pathways as an adjunct therapy may be beneficial for SMA^62,63^. As Klf15 is part of the GC-GR-KLF15-BCAA pathway, previously shown to be dysregulated in SMA muscle, it is possible that mifepristone treatment in animal models or SMA patients may modulate BCAA levels. Indeed, GCs can influence the activity of enzymes, particularly branched-chain keto acid dehydrogenase (BCKDH), involved in BCAA catabolism^3,64^. Therefore, antagonising GC signalling has the potential to impair BCAA breakdown, leading to higher circulating BCAA levels.

Our in vivo work was conducted in both vertebrate and invertebrate models of SMA, which led to several key observations such as the ability of mifepristone to improve the survival of *Smn*^*2B/-*^ mice as well as improve neuromuscular pathology in both *Smn*^*2B/-*^ mice and *C. elegans smn-1*(ok355) models. Our findings support previous work, from our group and others, that demonstrate that SMN-independent therapeutics alone can improve disease phenotypes, including lifespan^3,20,65^. In the current study, these improvements were seen when mifepristone was administered at the later time-points of P5 and P8 in *Smn*^*2B/-*^ mice, demonstrating that SMN-independent treatments can be administered later on during disease progression and still be able to attenuate pathology^66^. While *C. elegans* is a model organism, specifically in the context of genetics or aging, that often provides insights for further investigations in mice, there are also many apparent differences between both species^67^. Here, we demonstrated differences in their response to mifepristone treatment, particularly in terms of *Smn* levels, potentially due to differential regulation of the GC-GR-Klf15 pathway. Furthermore, mifepristone did not improve the survival of severe Taiwanese SMA mice or the SMA *C. elegans*, suggesting that peripheral pathologies may have a greater impact on disease phenotype in milder forms of mammalian SMA^66^. The adult population or patients with milder forms of SMA, including those now receiving therapy, may therefore benefit greatly from second-generation therapies^68^.

Given that a single dose of scAAV9-*SMN1* can significantly increase life expectancy, we combined scAAV9-*SMN1* with 500 μg/g mifepristone in *Smn*^*2B/-*^ mice^69^. While delivery of *SMN1* is sufficient to significantly improve lifespan in both mouse models and patients, this therapeutic approach does have limitations^49,70^. Indeed, as the longevity of this therapy is not yet known, it is possible that *SMN1* cDNA will become diluted over time in dividing cells, thus preventing prolonged peripheral expression ^71^. Furthermore, the amount of viral vector that can be delivered is currently limited due to the viral affinity for the liver^71^. In fact, research has shown mifepristone can control expression of transgenes that are potentially toxic, as an activator in drug-dependent inducible systems^72^. Ultimately, there is wide clinical evidence demonstrating the safety of mifepristone following both acute and chronic dosing regimens and most patients will likely benefit from a combinatorial approach to their treatment^71,73,74^.

In the current study, a combinatorial approach enabled us to determine any synergistic activities as well as evaluate whether we could expand the therapeutic benefit of scAAV9-*SMN1* to further improve pathology by targeting metabolic perturbations in *Smn*^*2B/-*^ mice. While scAAV9-*SMN1* combined with 500 μg/g mifepristone did not change the phenotypic outcomes of *Smn*^*2B/-*^ mice compared to gene therapy alone, molecular analyses of *Klf15* expression in tissues revealed differences between mifepristone-treated *Smn*^*2B/-*^ and *Smn*^*2B/*+^ mice. For instance, *Klf15* levels were significantly reduced in the liver of scAAV9-*SMN1* combined with 500 μg/g mifepristone-treated *Smn*^*2B/-*^ mice, but not *Smn*^*2B/*+^ animals that received the same combinatorial treatment. This suggests that mifepristone may preferentially target the liver when combined with an *SMN* rescuing therapy in a diseased state. As *Klf15* has been reported as a regulator of hepatic maturation, the decrease of *Klf15* in *Smn*^*2B/-*^ livers following combinatorial treatment may be a sign of improved liver development following rescue of liver pathology by *SMN1* restoration^75,76^. Additionally, mifepristone has been shown to reduce liver enzymes in patients with MASLD and could be beneficial knowing that scAAV9-*SMN1* can cause liver toxicity^46^. Therefore, short term administration of mifepristone (P5-21) in combination with scAAV9-*SMN1* likely induced long-lasting effects due to gene expression and protein synthesis changes. While this was not explored in the current study, early transcriptomic shifts may have led to persistent network-level alterations^77,78^. Such changes may have ongoing effects on cellular processes. Indeed, previous work suggests that short-term GR perturbations can lead to lasting DNA methylation and chromatin remodelling of regulated genes^79^.

Interestingly, we found an increase in myofiber area only in *Smn*^*2B/-*^ females following combinatorial treatment while there were no changes in males. This may be due to previously reported sex-dependent differences in both muscle metabolism and GC-GR activity^80–82^. Fundamentally, the beneficial effects of mifepristone have been associated with skeletal muscle pathology. We demonstrate that combinatorial therapy can improve certain aspects of disease pathology beyond that of treatment with the gene therapy alone in *Smn*^*2B/-*^ mice. As a result of its success so far, scAAV9-*SMN1* now requires long-term assessment as it is possible that patients living longer may experience muscle and metabolic pathologies that need to be addressed therapeutically^83^.

Metabolic pathologies in SMA have been reported, albeit to a limited extent, since before the genetic discovery of the *SMN* gene^4^. A more recent nutritional study found that nusinersen-treated patients still have gastrointestinal issues that can be modulated by an amino acid diet^18^. Additionally, therapies targeting complementary pathways that ultimately increase SMN in peripheral tissues prolong survival, demonstrating the importance of peripheral rescue beyond the canonical pathologies of SMA^84^. Along with the fact that the three approved gene therapies for SMA are unfortunately not cures for the disease, it is now clear that metabolic defects are often motor neuron-independent and should be autonomously addressed^85^.

Notably, there are many commonalities in metabolic pathologies between Cushing’s syndrome (CS), a serious endocrine disorder, and SMA, including hyperglycaemia, fatty liver and muscle atrophy. FDA-approved mifepristone (Corlux®) is currently a widely used therapy for CS. In addition, both respiratory and digestive system dysfunctions have been associated with the prevalence of depression in SMA patients^86^. Psychological disorders including anxiety and depression often accompany chronic disease and mifepristone has been investigated for its therapeutic benefit in patients with psychotic depression. Mifepristone-treated patients had reduced psychotic symptoms compared to placebo-treated patients, with a large safety margin^87^. Interestingly, amongst both patients with CS and psychotic disorders, mifepristone is known to attenuate an increase in weight^88–90^. Weight loss is most likely predominantly caused by antagonising the effects of cortisol and the knock-on influence this has on other metabolic processes in a disease state-dependent manner. While this would require further investigation, this could explain the decreased weight gain seen in *Smn*^*2B/*+^ mice. Ultimately, mifepristone could address metabolic pathologies, mental health issues and neural SMN-independent pathologies in SMA patients through its ability to cross the blood–brain barrier^91^.

In summary, our results along with our previous work^3^, suggest that the GC-GR-Klf15 pathway is dysregulated in metabolic tissues of SMA patients and mouse models and could play a role in glucose, lipid and amino acid metabolic dysfunctions^3^. Following ground-breaking research and clinical trials, the approval of three gene-directed therapies has revolutionised the field of SMA. With that said, it is now time to utilise these therapies to maximise potential therapeutic benefit. Additional peripheral and metabolic pathologies are important targets for therapeutic interventions that have until recently received little focus^4^. Future investigations should therefore be aimed at furthering our understanding of SMN-independent contributors to SMA pathology.

## Methods

### Cell culture

#### Cell proliferation and differentiation

C2C12 (generously provided by Professor Matthew JA Wood, University of Oxford) and 3T3-L1 (ATCC, Cat. #CL-173) cells were maintained in growth media consisting of Dulbecco’s Modified Eagle’s Media (DMEM) (Gibco, Cat. #2,041,859). FL83B cells (ATCC, Cat. #CRL-2390) were cultured in Kaighn’s modification of ham’s F-12 media (ATCC, Cat. #30–2004). All cells were supplemented with 10% FBS (Gibco, Cat. #2025814 K) and 1% penicillin/streptomycin (Gibco, Cat. #15,140,122). Cells were cultured at 37 °C with 5% CO_2_. C2C12 cells were differentiated in DMEM containing 1% FBS for 7 days. 3T3-L1 cells were differentiated in DMEM containing 1.0 μM dexamethasone, 0.5 mM methylisobutylxanthine (IBMX) (Sigma, Cat. #STBG0799V) and 1.0 μg/ml insulin (Sigma, Cat. #SLBW1822) for 48 h followed by DMEM containing 10% FBS and 1.0 μg/ml insulin for 10 days, replenished every 2–3 days.

#### Cell drug treatment

Dexamethasone (Merck, D4902-100 mg) and mifepristone (SLS, M8046-100 mg) were diluted in ethanol. Cells were treated with 1 μM, 5 μM and 10 μM dexamethasone for either 4, 8 or 24 h. Optimal concentration of dexamethasone was combined with mifepristone at 1 μM, 5 μM and 10 μM for varying time points dependent on preliminary experiments (4, 8 or 24 h).

#### Lactate dehydrogenase (LDH)-Glo™ cytotoxicity assay

Cell toxicity was determined using the Lactate dehydrogenase (LDH)-Glo™ assay kit (Promega) as per manufacturer’s instructions. Briefly, cells were treated with mifepristone (10 μM) and vehicle for either 24 or 72 h. Controls included no cell control, vehicle only cell control and maximum LDH release control. Max LDH control cells were exposed to 10% Triton X- 100 for 15 min. For all cell lines, a 1:300 dilution was used for undifferentiated cells and a 1:100 dilution was used for differentiated cells. The assay reaction was performed in the dark, at room temperature in 96-well opaque plates using 50 μl LDH detection reagent (1:200 reductase to detection enzyme mix) and 50 μl sample media (1:1). Luminescence was recorded after 60 min on a GloMax Explorer (Promega).

#### Bromo-2-deoxyuridine (BrdU) assay

Cell proliferation was determined by using 5- Bromo-2-deoxyuridine (BrdU) colorimetric system (Merck) as per manufacturer’s instructions. Briefly, cells were exposed to mifepristone (10 μM) or vehicle (ethanol) for 8 h prior to BrdU assay. The cells were labelled with BrdU (1:2000) for 16 h. Cells were exposed to an anti-BrdU fluorescence-labelled antibody. Using a spectrophotometric plate reader (GloMax Explorer (Promega)), BrdU absorbance was measured at dual wavelengths of 450–600 nm.

### Animals and procedures

#### Sex as a biological variable

Our study examined male and female animals, and depending on age and analyses, both similar findings are reported for both sexes and sex-dimorphic effects are reported.

#### Study approval

The *Smn*^*2B/-*^ mouse line was housed at the Keele University Biomedical Sciences Unit (BSU) and cared for according to Home Office Animal Scientific Procedures Act 1986 (ASPA) regulations (project license: P99AB3B95, personal license: IO376FCD7). All procedures were approved by the Keele University ethics committee (AWERB). Mouse models *Smn*^*2B/-*^*, Smn*^*2B/2B*^ mice (obtained from Charles River) and *Smn*^+*/-*^ mice (obtained from Jackson labs) were crossed to obtain *Smn*^*2B/-*^ and *Smn*^*2B/*+^ mice. *Smn*^+*/-*^ mice were crossed with *Smn*^+*/-*^ mice to obtain C57BL/6 J wild-type mice. Genotyping was performed on ear clips by PCR. Treatment groups were assigned at random, and animals of both sexes were used in all experiments.

The FVB.cg-*SMN1*tm1HungTg(*SMN2*)2Hung/J mice were crossed with *Smn*^+*/-*^ mice producing 50% SMA offspring (*Smn*^*-/-*^*;SMN2tg/0*) and 50% control carriers (*Smn*^*-*^^*/*+^*;SMN2tg/0*)^92^. These ‘Taiwanese’ SMA mice were housed in the University of Edinburgh animal facilities, in a 14-h/10 h light/ dark cycle in individually ventilated cages. All procedures were conducted according to Home Office ASPA 1986 regulations (Project License: PP1567597; PILs: IAC4805FD and 7E4CB171).

All animals were killed via the approved Schedule 1 method of exposure to carbon dioxide gas in a rising concentration, followed by confirmation of death via cervical dislocation or exsanguination.

The study is reported in accordance with ARRIVE guidelines (https://arriveguidelines.org) and the American Veterinary Medical Association​ (AVMA) Guidelines for the Euthanasia of Animals.

#### Drug administration in *Smn*^*2B/-*^* mice*

Mifepristone (RU486) (SLS, Cat. #M8046-100MG) was solubilised in 2 ml 0.5% carboxymethylcellulose (CMC) and sonicated for 3 min at 37 kHz. Mifepristone was administered by oral gavage at concentrations of 250 μg/g from post-natal day 8 (P8) or 500 μg/g starting from P5. The scAAV9-*SMN1* vector was produced by transient transfection of HEK293 cells adapted to suspension culture (HEKExpress™, ExcellGene SA) and the AAV9 particles were purified from the cell pellet and supernatant using affinity chromatography (POROS™ CaptureSelect™ AAV9 affinity resin; ThermoFisher Scientific)^93^. After concentration, the vector titer measured by dPCR was 1.4E13 VG/mL. The vector was administered by facial vein injections to post-natal P0 pups (1E11 VG/pup, 20 μl volume/pup). Combinatorial treatment with scAAV9-*SMN1* was administered by facial vein injections to P0 pups (1E11 VG/pup, 20 μl volume/pup) combined with 500 μg/g mifepristone by daily oral gavage starting at P5 until P21.

Phenotypic analysis of weight and righting reflex was conducted daily on all mice. Survival analyses were conducted on litters until defined humane endpoints were reached. For all experiments litters, containing males and females, were randomly assigned treatment. Triceps, tibialis anterior (TA), liver and BAT were harvested for molecular and histological analysis from *Smn*^*2B/-*^ mice and *Smn*^*2B/*+^ healthy littermates.

#### Drug administration in Taiwanese *Smn*^*-/-*^*;SMN2 mice*

Mifepristone was prepared as above. Phenotypic analyses were conducted daily on *Smn*^*-/-*^*;SMN2* mice. Litters undergoing daily 500 μg/g mifepristone treatment versus vehicle control were administered the drug on P3 by oral gavage up until animals reached their humane endpoint.

Litters undergoing daily 500 μg/g mifepristone treatment were administered the drug on P3 by oral gavage up until animals reached their humane endpoint. Procedures were performed in a laminar flow hood in the animal facility.

#### *C. elegans* SMA model

The LM99 *smn-1*(ok355)/*hT2* strain, segregates into homozygotes *smn-1*(ok355), lethal homozygotes *hT2/hT2*, and heterozygotes *smn-1(ok355)/hT2*. Homozygotes *smn-1*(ok355) resemble a severe SMA model. Heterozygotes smn-1/hT2 were used as controls. PHX8399 strain was generated by SunyBiotech. PHX8399 *(smn-1(ok355) I/hT2[smn-1(syb8399))* carries the endogenous wild type copy of *smn-1* tagged with wrmScarlet using CRISPR/Cas9-mediated genome editing. The strain was backcrossed two times (DIM51). Wild type animals were obtained from the HA1981 *(* +*)/hT2* strain which carries the *hT2* balancer chromosome to ensure a common genetic background. These animals were maintained at 20◦C on Nematode Growth Medium (NGM) plates seeded with Escherichia coli OP50 bacteria^94^.

Mifepristone (RU486) (SLS, Cat. #M8046-100MG) was dissolved in DMSO and added to the NGM agar solution at concentrations of 0, 1, 15 and 30 μM. Mifepristone was administered by to *C. elegans* by raising the animals on plates containing the vehicle or drug.

Neuromuscular assays were performed on *C. elegans* that were 3 days old. The pharyngeal pumping assay was performed as previously described^39^. Notably, an Axio Cam ICc5 camera on a Discovery V8 SteREO microscope was used for both movement assays. The pharyngeal pumping assay was filmed using 150X objective at 175 frames/10 s. A grinder movement in any axis was defined as a pumping event. Pumps were manually counted using Zen Pro software v2.3. Locomotion assays were filmed using a 63X objective at 15 frames/second. Mobility forward time, for 5 min, was quantified using WormLab 1.1 software (MBF Bioscience).

Lifespan assays were performed on age-matched *C. elegans*. Animals were reared on plates containing either the vehicle or drug on day 3. Animals were scored daily at 20 °C and censored for loss, bagging, internal hatching of progeny or climbing out of the plate. Each condition included ≥ 90 animals and was repeated in 3 independent trials.

#### Laminin staining of skeletal muscle

TA muscles were fixed in 4% paraformaldehyde (PFA) overnight. Tissues were mounted in cryomoulds and quickly frozen in liquid nitrogen. Tissues were sliced at 13 μM and stored at − 20 °C. Briefly, sections were dipped in acetone for 5 min and left to air dry for 30 min and incubated for 2 h in blocking buffer (0.3% triton X, 20% FBS, 20% BSA in PBS). Samples were incubated overnight at 4 °C with a rat anti-laminin antibody (1:1000, L0663, Sigma Aldrich) in blocking buffer. Followed by goat-anti-rat IgG 488 secondary antibody (1:250, AlexaFluor488, ThermoFisher scientific) for one hour. Tissues were mounted in media containing DAPI (SLS, F6057-20ML). Images were taken with a fluorescence microscope. TA muscle fibre area was measured on at least100 fibres from 3 to 5 sections per animals using Fiji. Images were assigned a random ID number by another experimenter and true IDs were only revealed once quantification was finalised.

#### Oil-red-O staining of mouse liver

Liver tissues were flash frozen and stored at – 80 °C. For sectioning, tissues were embedded in a PolyFreeze solution containing 30% glucose using in liquid nitrogen. Sections (10 µm) were cut using a cryostat. The Oil Red O stock solution was prepared by dissolving 0.5 g Oil Red O powder in 100 mL of isopropanol, mixed thoroughly and left overnight to ensure complete dissolution. Slides were briefly rinsed in deionized water (ddH_2_O), incubated in 60% isopropanol for 2 min and stained with 0.5% Oil Red O for 15 min. After staining, slides were washed in 60% isopropanol for 2 min, followed by a 3-min rinse in ddH_2_O. Slides were mounted with aqueous DAPI-containing medium. Images were captured using a Nikon DS-U2 light microscope with a 10X objective.

#### Primary type III SMA human deltoid myoblasts

Dr Stephanie Duguez (Ulster university) generously provided cell pellets and/or RNA samples from SMA Type III primary human myoblasts and age-matched healthy controls obtained from deltoid muscle biopsies.

#### qPCR

RNA was extracted from cells using the ISOLATE II RNA Mini Kit (Bioline), following manufacturer’s instructions. Tissues (triceps, BAT and liver) were homogenised in RLT buffer using 7 mm stainless steel balls (Qiagen) and the Tissue Lyser LT (Qiagen) set at 50 oscillations for 2 min. Extraction of RNA from skeletal muscle was conducted using the RNeasy Fibrous tissue kit (Qiagen). The ISOLATE II RNA mini kit (Qiagen) was used to extract RNA from liver, while BAT RNA was extracted using the RNeasy lipid tissue mini kit (Qiagen), as per manufacturer’s instructions. A Nanodrop 1000 spectrophotometer (ThermoScientific) was used to measure the RNA concentrations (ug/ul) of samples alongside a blank control sample using RNase-free water. cDNA was prepared using cDNA synthesis mix (4 μl) and 20 × RTase (1 μl). cDNA was then produced by reverse transcription using a^3^Prime Thermocycler (Techne). qPCR was performed on StepOnePlus™ Real-time PCR system (ThermoFisher Scientific). *PolJ* was used as a housekeeping gene and relative gene expression was quantified using the Pfaffl method and primer efficiency was calculated using LinRegPCR V11.0 software.

For *C. elegans*, total RNA was isolated from synchronised populations of *C. elegans* using TRIzol reagent, following standard protocols. Extracted RNA samples underwent cDNA synthesis using the Invitrogen™ SuperScript™ III First-Strand Synthesis System. The expression of the *csq-1* housekeeping gene was used to normalise gene expression.

The list of mouse, human and nematode primers used can be found in Supplementary Table 1.

#### Smn quantification in *C. elegans*

For Smn-1 quantification in the *C. elegans* model, the wild type copy of *smn-1* on the *hT2* balancer was tagged with wrmScarlet to the C-terminus (Ex 569 nm, Em 594 nm). Briefly, animals were immobilised with 3% 2,3-butanedione monoxime (BDM) and mounted on to 2% agar pads. Animals were imaged using a Zeiss AXIO imager M.2 at 10 × magnification with a Texas Red filter. Whole body fluorescence intensity was measured using the ZEN 2 (Blue Edition) 3.1 software. Each condition included ≥ 25 animals and was repeated in 3 independent trials.

### Statistical analysis

The most up to date GraphPad Prism software was used for data analysis and are presented as the mean ± standard error the mean. Appropriate statistical tests were used depending on data set, including unpaired t-test, one- way analysis of variance (ANOVA) and two-way ANOVA followed by post-hoc tests. Kaplan–Meier survival analysis was performed using the log-ran (Mantel-Cox) test. Statistical significance was found with P values less than 0.05 displayed as *P < 0.05, **P < 0.01, ***P < 0.001, ****P < 0.0001. Outliers were identified using the GraphPad Grubb’s Test Individual data sets were all ran through the Grubbs’ test calculator, with the significance level set to alpha 0.05, to determine whether the most extreme value in the data set was a notable outlier compared to the other values. If this was the case, outliers were excluded from the analysis.

## Supplementary Information

Supplementary Information.

## Data Availability

All data associated with this study are available in the main text or supplementary materials. Raw data can be provided upon request. Please contact Professor Bowerman (m.bowerman@keele.ac.uk) for any data request from this study.
