# Supplementary material for: Mifepristone alone and in combination with scAAV9-SMN1 gene therapy improves disease phenotypes in Smn2B/- spinal muscular atrophy mice
Source: Sci Rep. 2025 Nov 17;15:40225. doi: 10.1038/s41598-025-24050-3 (PMC12623908; doi:10.1038/s41598-025-24050-3)
Supplement: Supplementary file 1 — Supplementary Information. [file 41598_2025_24050_MOESM1_ESM.pdf]

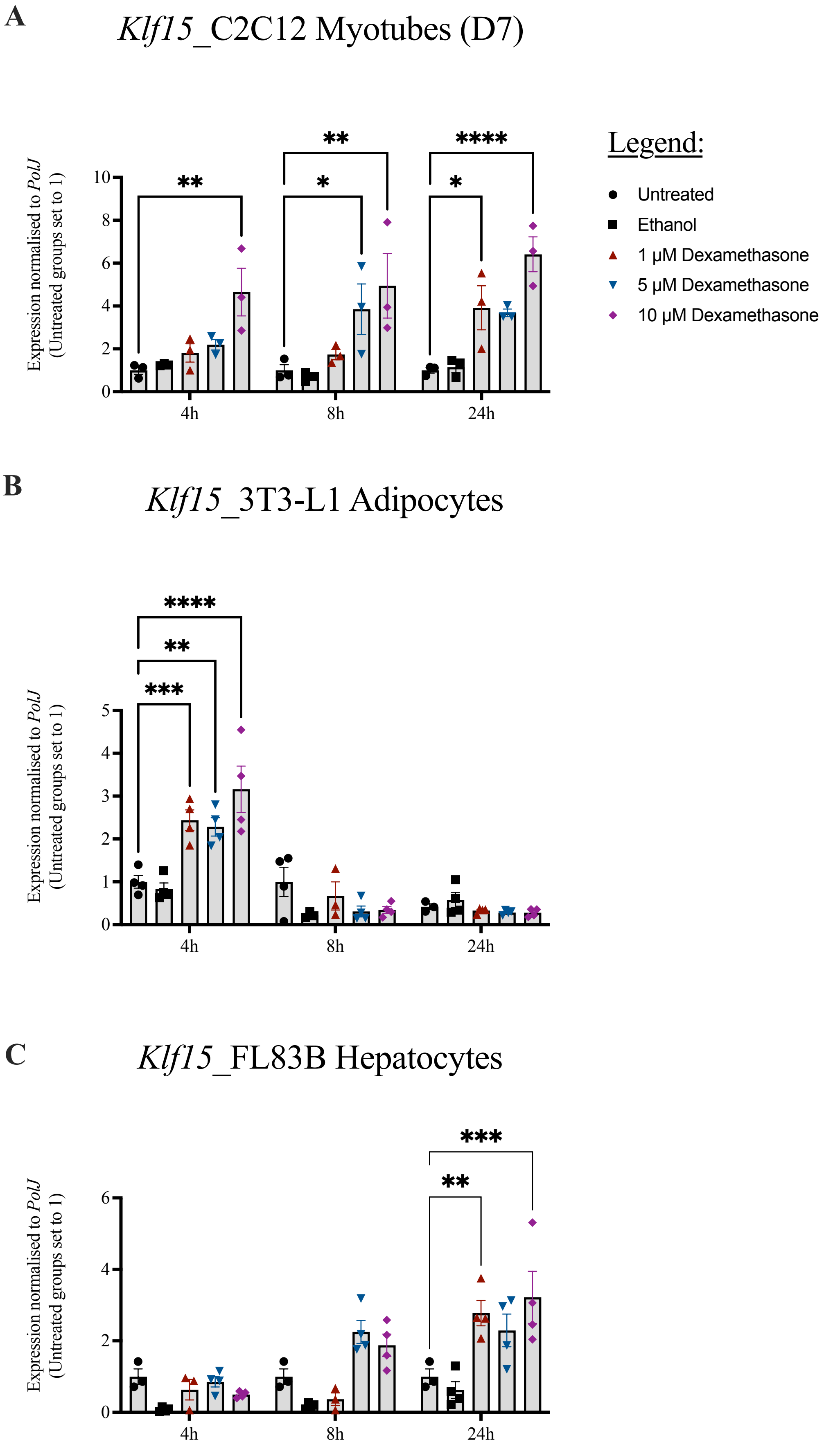
A
Klf15_C2C12 Myotubes (D7)
Expression normalised to PolJ
(Untreated groups set to 1)
0
2
4
6
8
10
**
*
**
*
****
4h
8h
24h
Legend:
Untreated
Ethanol
1 µM Dexamethasone
5 µM Dexamethasone
10 µM Dexamethasone
B
Klf15_3T3-L1 Adipocytes
Expression normalised to PolJ
(Untreated groups set to 1)
0
1
2
3
4
5
****
**
***
4h
8h
24h
C
Klf15_FL83B Hepatocytes
Expression normalised to PolJ
(Untreated groups set to 1)
0
2
4
6
***
**
4h
8h
24h

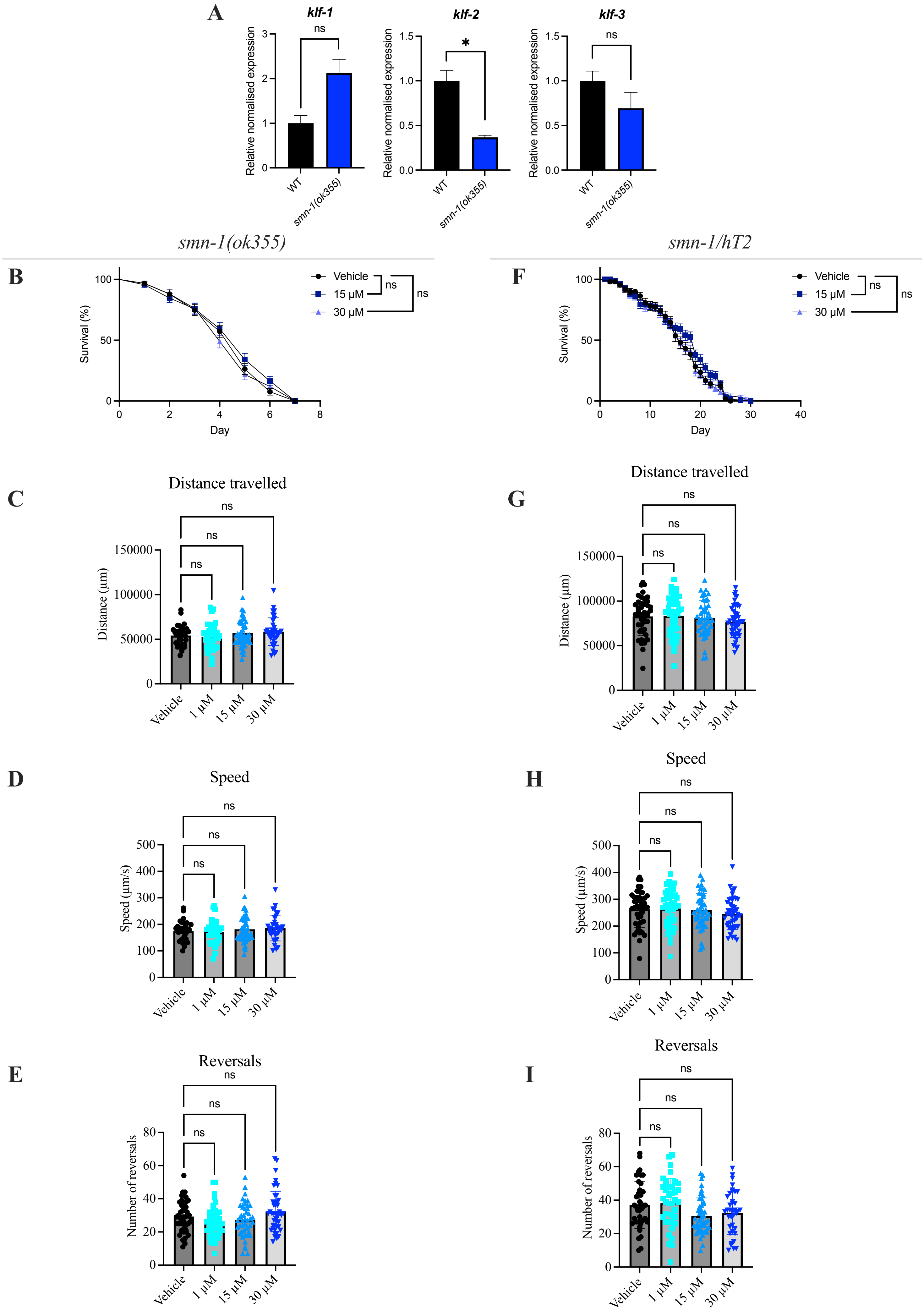

A
klf-1
klf-2
klf-3
Relative normalised expression
ns
*
WT
smn-1(ok355)
smn-1(ok355)
smn-1/hT2
B
F
Survival (%)
Day
Vehicle
15 µM
30 µM
C
G
Distance travelled
Distance (µm)
D
H
Speed
Speed (µm/s)
E
I
Reversals
Number of reversals
Vehicle
1 µM
15 µM
30 µM

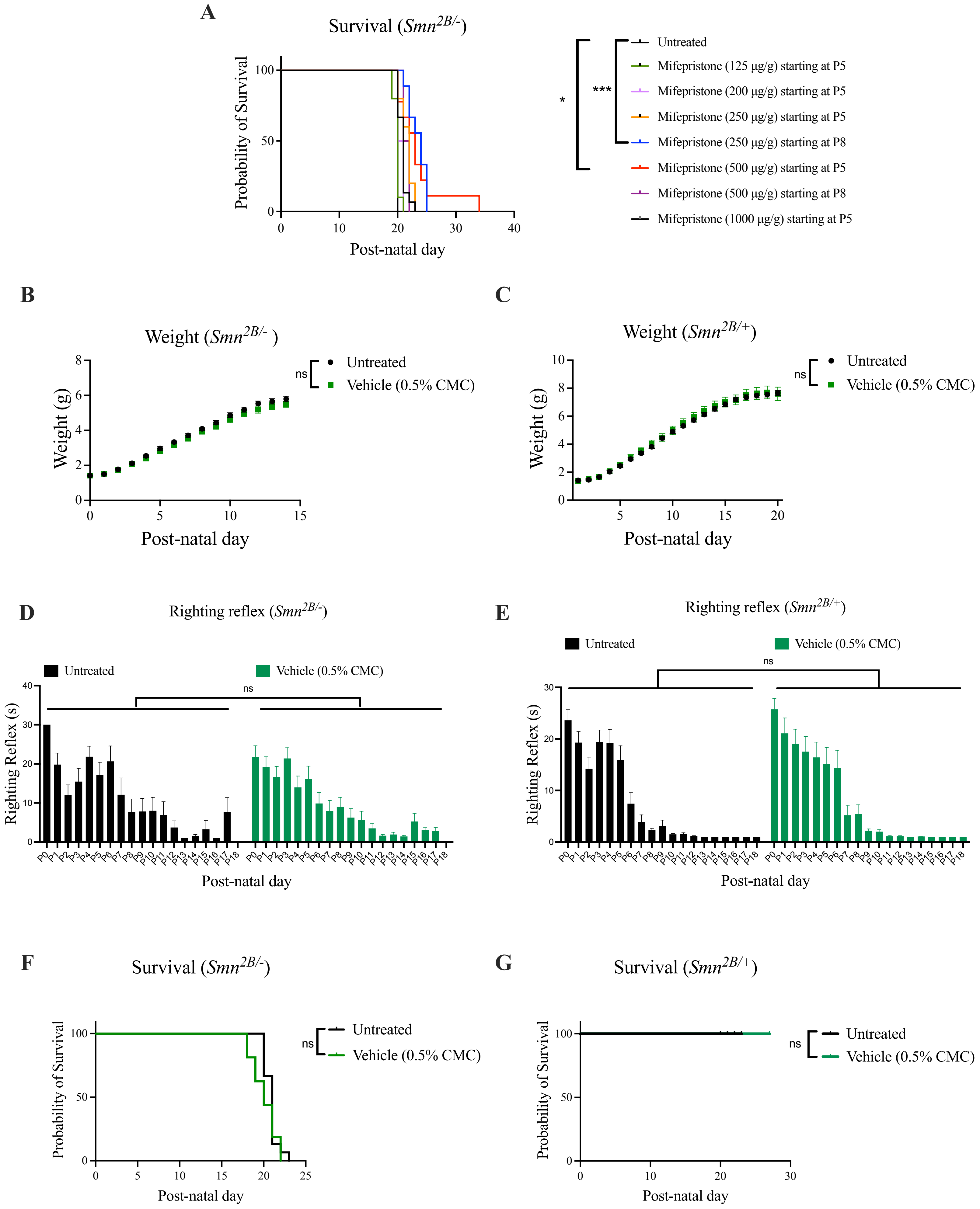
A
Survival (Smn2B/-)
Probability of Survival
Post-natal day
Untreated
Mifepristone (125 µg/g) starting at P5
Mifepristone (200 µg/g) starting at P5
Mifepristone (250 µg/g) starting at P5
Mifepristone (250 µg/g) starting at P8
Mifepristone (500 µg/g) starting at P5
Mifepristone (500 µg/g) starting at P8
Mifepristone (1000 µg/g) starting at P5
*
***
B
Weight (Smn2B/-)
Weight (g)
Post-natal day
ns
Untreated
Vehicle (0.5% CMC)
C
Weight (Smn2B/+)
Weight (g)
Post-natal day
ns
Untreated
Vehicle (0.5% CMC)
D
Righting reflex (Smn2B/-)
Untreated
Vehicle (0.5% CMC)
ns
Righting Reflex (s)
Post-natal day
E
Righting reflex (Smn2B/+)
Untreated
Vehicle (0.5% CMC)
ns
Righting Reflex (s)
Post-natal day
F
Survival (Smn2B/-)
Probability of Survival
Post-natal day
ns
Untreated
Vehicle (0.5% CMC)
G
Survival (Smn2B/+)
Probability of Survival
Post-natal day
ns
Untreated
Vehicle (0.5% CMC)

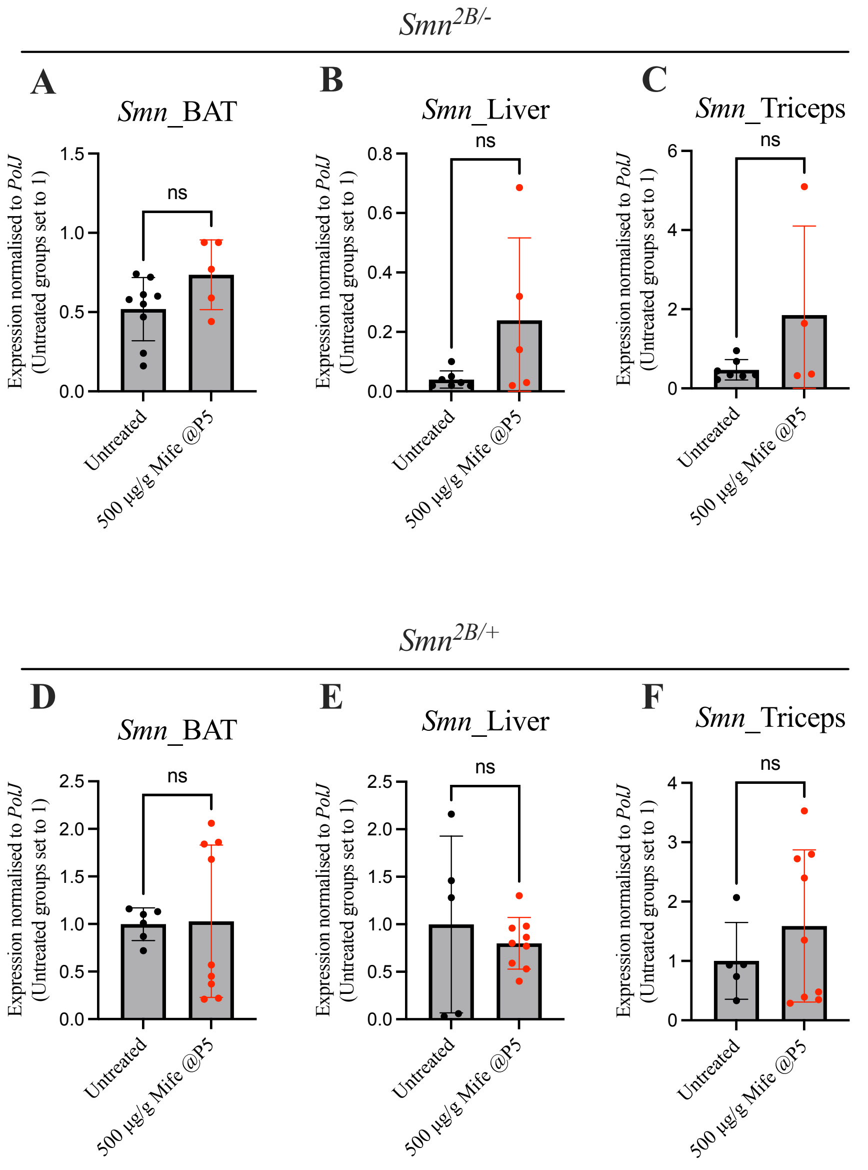

Smn2B/-
A
Smn_BAT
Expression normalised to PolJ (Untreated groups set to 1)
1.5
1.0
0.5
0.0
ns
Untreated
500 µg/g Mife @P5
B
Smn_Liver
0.8
0.6
0.4
0.2
0.0
C
Smn_Triceps
6
4
2
0
Smn2B/+
D
2.5
2.0
1.5
1.0
0.5
0.0
E
F
4
3
2
1
0

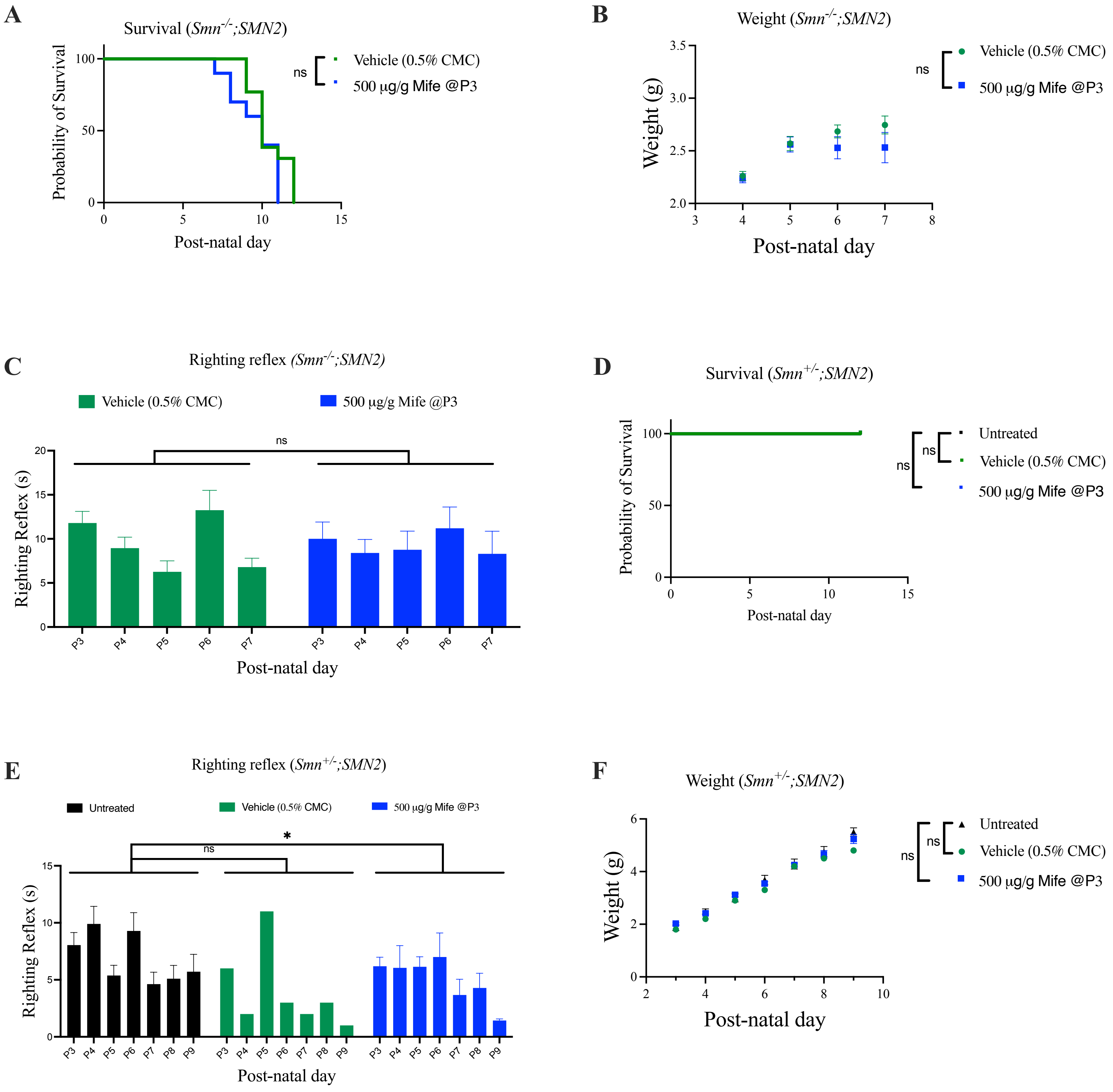
A
Survival (Smn-/-;SMN2)
Probability of Survival
Post-natal day
ns
Vehicle (0.5% CMC)
500 µg/g Mife @P3
B
Weight (Smn-/-;SMN2)
Weight (g)
Post-natal day
ns
Vehicle (0.5% CMC)
500 µg/g Mife @P3
C
Righting reflex (Smn-/-;SMN2)
Vehicle (0.5% CMC)
500 µg/g Mife @P3
ns
Righting Reflex (s)
Post-natal day
D
Survival (Smn+/-;SMN2)
Probability of Survival
Post-natal day
Untreated
Vehicle (0.5% CMC)
500 µg/g Mife @P3
ns
ns
E
Righting reflex (Smn+/-;SMN2)
Untreated
Vehicle (0.5% CMC)
500 µg/g Mife @P3
*
ns
Righting Reflex (s)
Post-natal day
F
Weight (Smn+/-;SMN2)
Weight (g)
Post-natal day
Untreated
Vehicle (0.5% CMC)
500 µg/g Mife @P3
ns
ns

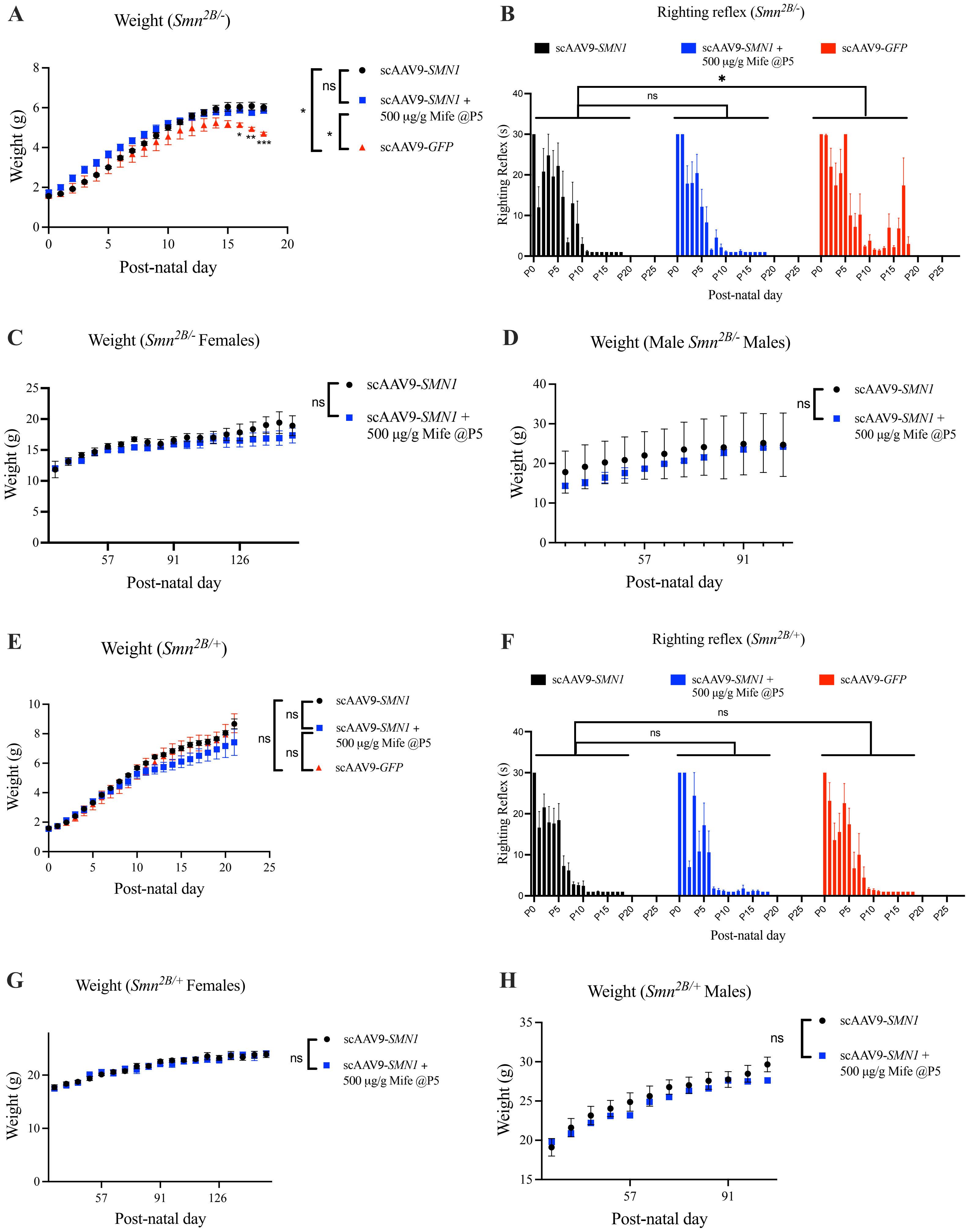

A
Weight (Smn2B/-)
scAAV9-SMN1
scAAV9-SMN1 + 500 µg/g Mife @P5
scAAV9-GFP
ns
*
Weight (g)
Post-natal day
B
Righting reflex (Smn2B/-)
Righting Reflex (s)
Post-natal day
C
Weight (Smn2B/- Females)
D
Weight (Male Smn2B/- Males)
E
Weight (Smn2B/+)
F
Righting reflex (Smn2B/+)
G
Weight (Smn2B/+ Females)
H
Weight (Smn2B/+ Males)

Supplementary Table 1. List of qPCR primers used.

Gene	Forward primer	Reverse primer
PolJ (mouse)	ACCACACTCTGGGGAACA TC	CTCGCTGA TGAGGTCTGTGA
GRα (mouse)	AAAGAGCTAGGAAAAGCCATTGTC	TCAGCTAACATCTCTGGGAATTCA
GRβ (mouse)	AAAGAGCTAGGAAAAGCCATTGTC	CTGTCTTTGGGCTTTTGAGATAGG
Klf15 (mouse)	TGCGTCGGCACACAGGCGAGAA	CCGGTGCCTTGACAACTCA TCT
HKII (mouse)	GAAGGGGCTAGGAGCTACCA	CTCGGAGCACACGGAAGTT
Glut4 (mouse)	GACGGACACTCCATCTGTTG	CATAGCTCATGGCTGGAACC
PGC1α (mouse)	TGGAGTGACATAGAGTGTGCTGC	CTCAAATATGTTCGCAGGCTCA
Smn (mouse)	TGCTCCGTGGACCTCATTTCTT	TGGCTTTCCTGGTCCTAATCCTGA
Pol2RA (human)	CAACGCACACATCCAGAACG	TCCTTGACTCCCTCCACCAC
Klf15 (human)	GCTTGAGTTAAATGTGCAGGG	TTCTAAATCAGGGTTGGGAGG
Klf-1 (nematode)	GAGCCTTTTCCAACCGACAAC	CTGGAGCGAACGCACTTTTC
Klf-2 (nematode)	GTAAAGACGAGGAAGATCCCAGG	GACCTTGCCGCAACCTTGA
Klf-3 (nematode)	CTCCAAGATATGAAGAAGACTGGGC	TGGTGGAAGAAGCATCCTTGG
Csq-1 (nematode)	GCCTTGCGCTAGTGGTTGTGC	GCTCTGAGTCGTCCTCTTCCACG

SUPPLEMENTARY FIGURE LEGENDS

Supplementary Figure 1. Dexamethasone-induced *Klf15* expression in C2C12 (muscle), 3T3-L1 (adipose) and FL83B (liver) cells is time- and dose-dependent. A, *Klf15* expression in differentiated C2C12 myotubes (D7) treated with dexamethasone (1, 5 or 10 μM) for 4, 8 or 24 hours. Data are mean ± SEM, N = 3-4 experimental repeats (3-4 wells/repeat), two-way ANOVA, ns = non-significant, **P*<0.05, ***P*<0.01, *****P*<0.0001. **B**, *Klf15* expression in 3T3-L1 adipocytes cells treated with dexamethasone (1, 5 or 10 μM) for 4, 8 or 24 hours. Data are mean ± SEM, N = 3-4 experimental repeats (3-4 wells/repeat), two-way ANOVA, ***P*<0.01, ****P*<0.001, *****P*<0.0001. **C**, *Klf15* expression in FL83B hepatocyte cells treated with dexamethasone (1, 5 or 10 μM) for 4, 8 or 24 hours. Data are mean ± SEM, N = 3-4 experimental repeats (3-4 wells/repeat), two-way ANOVA, ***P*<0.01, ****P*<0.001.

Supplementary Figure 2. Mifepristone did not affect survival, distance travelled, speed or reversals in a severe SMA *C. elegans smn-1* (*ok355*) model. A, *Klf-1* (F56F11.3) *Klf-2* (F53F8.1) and *Klf-3* (F54H5.4) expression in control *C. elegans smn-1/hT2* compared to SMA *C. elegans smn-1* (ok355). Data are mean ± SEM, N = 2-3 animals per experimental group, t-test, **P*<0.05, ns = not significant. **B**, Lifespan assay in vehicle- or mifepristone-treated (15 or 30 μM) age-matched SMA *C. elegans smn-1* (ok355). N ≥ 90 animals per experimental group, Kaplan-Meier survival curves, Log-rank (Mantel-Cox) test, ns = not significant. **C-E,** Distance travelled (C), speed (D) and reversals (E) in vehicle- or mifepristone-treated (1, 15 or 30 μM) SMA *C. elegans smn-1* (ok355). Data are mean ± SEM, N = 25 animals per experimental group, one-way ANOVA, ns = not significant. **F**, Lifespan assay in vehicle or mifepristone-treated (15 or 30 μM) age-matched control *C. elegans smn-1/hT2*. N≥90 animals per experimental group, Kaplan-Meier survival curves, Log-rank (Mantel-Cox) test, ns = not significant. **G-I,** Distance travelled (G), speed (H) and reversals (I) in vehicle- or mifepristone-treated (1, 15 or 30 μM) *C. elegans smn-1/hT2*.

Data are mean ± SEM, N = 25 animals per experimental group, one-way ANOVA, ns = not significant.

Supplementary Figure 3. Mifepristone dose optimisation and evaluation vehicle treatment in *Smn*$^{2B/-}$ and *Smn*$^{2B/+}$ mice. A, Survival curves of untreated and mifepristone-treated (125-1000 µg/g starting at P5 or P8) *Smn*$^{2B/-}$ mice. Data are Kaplan-Meier survival curves, N = 9-14 animals per experimental group, Log-rank (Mantel-Cox) test, **P*<0.05, ****P*<0.001. Survival data for optimal doses selected (250 µg/g and 500 µg/g) are repeated in figure 2C. **B**, Daily weights of untreated and vehicle-treated (0.5% carboxymethylcellulose (CMC)) *Smn*$^{2B/-}$ mice. Data are mean ± SEM, N = 9-14 animals per experimental group, two-way ANOVA, ns = not significant. **C**, Daily weights of untreated and vehicle-treated (0.5% CMC) *Smn*$^{2B/+}$ mice. Data are mean ± SEM, N = 9-14 animals per experimental group, two-way ANOVA, ns = not significant. **D**, Daily righting reflex of untreated and vehicle-treated (0.5% CMC) *Smn*$^{2B/-}$ mice. Data are mean ± SEM, N = 9-14 animals per experimental group, one-way ANOVA, ns = not significant. **E**, Daily righting reflex of untreated and vehicle-treated (0.5% CMC) *Smn*$^{2B/+}$ mice. Data are mean ± SEM, N = 9-14 animals per experimental group, one-way ANOVA, ns = not significant. **F**, Survival curves of untreated and vehicle-treated (0.5% CMC) *Smn*$^{2B/-}$ mice. Data are Kaplan-Meier survival curves, N = 9-14 animals per experimental group, Log-rank (Mantel-Cox) test, ns = not significant. **G**, Survival curves of untreated and vehicle-treated (0.5% CMC) *Smn*$^{2B/+}$ mice. Data are Kaplan-Meier survival curves, N = 9-14 animals per experimental group, Log-rank (Mantel-Cox) test, ns = not significant.

Supplemental Figure 4. Mifepristone does not impact *Smn* expression in *Smn*$^{2B/-}$ and *Smn*$^{2B/+}$ mice. A-C, BAT *Smn* expression in BAT (A), liver (B) and triceps (C) from post-natal day (P) 18 untreated and 500 µg/g mifepristone-treated *Smn*$^{2B/-}$ mice. Data are mean ± SEM, N = 5-9 animals per experimental group, unpaired *t-test*, ns = not significant. **D-F,** *Smn* expression in BAT (D), E (liver) and triceps (F) from P18 untreated and 500 µg/g mifepristone-treated *Smn*$^{2B/+}$ mice.

Data are mean ± SEM, N = 5-9 animals per experimental group, unpaired *t-test*, ns = not significant.

Supplementary Figure 5. Mifepristone does not improve disease phenotypes in severe *Smn*$^{-/-}$*;SMN2* SMA mice. Mifepristone (500 µg/g) was administered daily, starting at post-natal day (P) 3, by oral gavage suspended in 0.5% carboxymethylcellulose (CMC). **A**, Survival curves of vehicle- and mifepristone-treated severe *Smn*$^{-/-}$*;SMN2* SMA mice. Data are Kaplan-Meier survival curves, N = 10-13 animals per experimental group, Log-rank (Mantel-Cox) test, ns = not significant. **B**, Daily weights of vehicle- and mifepristone-treated severe *Smn*$^{-/-}$*;SMN2* SMA mice. Data are mean ± SEM, N = 10-13 animals per experimental group, two-way ANOVA, ns = not significant. **C**, Daily righting reflex of vehicle- and mifepristone-treated severe *Smn*$^{-/-}$*;SMN2* SMA mice. Data are mean ± SEM, N = 10-13 animals per experimental group, one-way ANOVA, ns = not significant. **D**, Survival curves of vehicle- and mifepristone-treated *Smn*$^{+/-}$*;SMN2* healthy control mice. Data are Kaplan-Meier survival curves, N = 10-13 animals per experimental group, Log-rank (Mantel-Cox) test, ns = not significant. **E**, Daily righting reflex of vehicle- and mifepristone-treated *Smn*$^{+/-}$*;SMN2* healthy control mice. Data are mean ± SEM, N = 10-13 animals per experimental group, one-way ANOVA, ns = not significant, $^{*}P<0.05$. **F**, Daily weights of vehicle- and mifepristone-treated *Smn*$^{+/-}$*;SMN2* healthy control mice. Data are mean ± SEM, N = 10-13 animals per experimental group, two-way ANOVA, ns = not significant.

Supplementary Figure 6. No significant differences in weight, righting reflex and survival in *Smn*$^{2B/-}$ and *Smn*$^{2B/+}$ mice treated with scAAV9-*SMN1* or scAAV9-*SMN1* + mifepristone. scAAV9-*SMN1* or scAAV9-*GFP* was administered at post-natal (P) 0 by a facial intravenous injection (IV) and mifepristone (500 µg/g) from P5 to P21 by oral gavage. **A**, Daily weights of scAAV9-*GFP*-, scAAV9-*SMN1*- or scAAV9-*SMN1* + mifepristone-treated *Smn*$^{2B/-}$ SMA mice. Data are mean ± SEM, N = 10-13 animals per experimental group, two-way ANOVA, $^{*}P<0.05$, $^{**}P<0.01$, $^{***}P<0.001$. **B**, Daily righting reflex of scAAV9-*GFP*-, scAAV9-*SMN1*- or scAAV9-*SMN1* + mifepristone-treated *Smn*$^{2B/-}$ SMA mice. Data are mean ± SEM, N = 10-13 animals per

experimental group, one-way ANOVA, ns = not significant, **P*<0.05. **C**, Weekly weights of scAAV9-*SMN1*- or scAAV9-*SMN1* +mifepristone-treated *Smn*$^{2B/-}$ SMA females. Data are mean ± SEM, N = 10-13 animals per experimental group, two-way ANOVA, ns = not significant. **D**, Weekly weights of scAAV9-*SMN1*- or scAAV9-*SMN1* +mifepristone-treated *Smn*$^{2B/-}$ SMA males. Data are mean ± SEM, N = 10-13 animals per experimental group, two-way ANOVA, ns = not significant. **E**, Daily weights of scAAV9-*GFP*-, scAAV9-*SMN1*- or scAAV9-*SMN1* +mifepristone-treated *Smn*$^{2B/+}$ healthy control mice. Data are mean ± SEM, N = 10-13 animals per experimental group, two-way ANOVA, ns = not significant. **F,** Daily righting reflex of scAAV9-*GFP*-, scAAV9-*SMN1*- or scAAV9-*SMN1* + mifepristone-treated *Smn*$^{2B/+}$ healthy control mice. Data are mean ± SEM, N = 10-13 animals per experimental group, one-way ANOVA, ns = not significant. **G**, Weekly weights of scAAV9-*SMN1*- or scAAV9-*SMN1* + mifepristone-treated *Smn*$^{2B/+}$ healthy control females. Data are mean ± SEM, N = 10-13 animals per experimental group, two-way ANOVA, ns = not significant. **H**, Weekly weights of scAAV9-*SMN1*- or scAAV9-*SMN1* + mifepristone-treated *Smn*$^{2B/+}$ healthy control males. Data are mean ± SEM, N = 10-13 animals per experimental group, two-way ANOVA, ns = not significant.

SUPPLEMENTARY TABLES

Supplementary Table 1. List of qPCR primers used.
